# Arginine Catabolism and Polyamine Biosynthesis Pathway Disparities Within *Francisella tularensis* Subpopulations

**DOI:** 10.3389/fmicb.2022.890856

**Published:** 2022-06-20

**Authors:** Yinshi Yue, Bhanwar Lal Puniya, Tomáš Helikar, Benjamin Girardo, Steven H. Hinrichs, Marilynn A. Larson

**Affiliations:** ^1^Department of Pathology and Microbiology, University of Nebraska Medical Center, Omaha, NE, United States; ^2^Department of Biochemistry, University of Nebraska–Lincoln, Lincoln, NE, United States

**Keywords:** *Francisella tularensis*, tularemia, metabolism, amino acid metabolism, polyamine biosynthesis

## Abstract

*Francisella tularensis* is a highly infectious zoonotic pathogen with as few as 10 organisms causing tularemia, a disease that is fatal if untreated. Although *F. tularensis* subspecies *tularensis* (type A) and subspecies *holarctica* (type B) share over 99.5% average nucleotide identity, notable differences exist in genomic organization and pathogenicity. The type A clade has been further divided into subtypes A.I and A.II, with A.I strains being recognized as some of the most virulent bacterial pathogens known. In this study, we report on major disparities that exist between the *F. tularensis* subpopulations in arginine catabolism and subsequent polyamine biosynthesis. The genes involved in these pathways include the *speHEA* and *aguAB* operons, along with *metK*. In the hypervirulent *F. tularensis* A.I clade, such as the A.I prototype strain SCHU S4, these genes were found to be intact and highly transcribed. In contrast, both subtype A.II and type B strains have a truncated *speA* gene, while the type B clade also has a disrupted *aguA* and truncated *aguB*. Ablation of the chromosomal *speE* gene that encodes a spermidine synthase reduced subtype A.I SCHU S4 growth rate, whereas the growth rate of type B LVS was enhanced. These results demonstrate that spermine synthase SpeE promotes faster replication in the *F. tularensis* A.I clade, whereas type B strains do not rely on this enzyme for *in vitro* fitness. Our ongoing studies on amino acid and polyamine flux within hypervirulent A.I strains should provide a better understanding of the factors that contribute to *F. tularensis* pathogenicity.

## Introduction

*Francisella tularensis* is a highly infectious pathogen with as few as 10 organisms causing the fatal zoonotic disease tularemia if left untreated, and infects over 250 species, including humans, rodents, lagomorphs, and arthropods (Sjostedt, 2007). This highly virulent and easily aerosolized facultative intracellular pathogen is classified as a Tier 1 category A select agent by the Centers for Disease Control and Prevention (CDC; Bhattacharjee, 2011). Although the subspecies and subtypes within *F. tularensis* share over 99.5% average nucleotide identity, major disparities in virulence exist between these subpopulations. These pathogenicity differences are due in part to genomic rearrangements, which are primarily due to the transposition of insertion sequence (IS) elements and direct repeats, that affect gene integrity and expression (Larson et al., 2011, 2015). IS elements typically encode transposases that are flanked by small inverted repeats (Mahillon and Chandler, 1998). As has been observed for other highly virulent intracellular pathogens, *F. tularensis* genomes are small (<2 Mbp) and contain numerous IS elements with IS*Ftu1* being the most abundant mobile element (Table 1; Rohmer et al., 2007; Larson et al., 2015). Consequently, IS*Ftu1* is often responsible for the observed gene disruptions that result in a pseudogene, and nucleotide polymorphisms can create a premature stop codon that produce a truncated and non-functional gene product (Rohmer et al., 2007; Carlson et al., 2009).

**Table 1:** Genomic features in representative strains from each *Francisella tularensis* subpopulation.

*Francisella tularensis* features^a^	A.I	A.I	A.II	A.II	B	B
	SCHU S4 (1941, Ohio)^b^	MA00-2987 (2000, Massachusetts)^b^	WY96-3418 (1996, Wyoming)^b^	WY-00W4114 (2000, Wyoming)^b^	FSC200 (1998, Sweden)^b^	LVS (1930, Russia)^b^
Length (bp)	1,892,775	1,892,645	1,898,476	1,899,252	1,894,157	1,895,994
GC content (%)	32.3	32.3	32.3	32.3	32.2	32.2
Total protein ORFs	1,660	1,773	1,761	1,757	1,604	1,689
Disrupted ORFs/pseudogenes	188	124	125	133	276	238
Large duplicated regions (>5 kbp)	3	3	3	3	3	3
*Francisella* pathogenicity islands	2	2	2	2	2	2
IS*Ftu1* transposase/IS elements^c^	47	47	48	48	58	59
All IS elements (full-length and remnants)^d^	74	74	101	101	107	109
Structural tRNA	38	38	38	38	38	38
Structural rRNA	10	10	10	10	10	10
Noncoding RNA	4	4	4	4	4	4

a Features were based on NCBI annotations for SCHU S4 NC_006570.2, MA00-2987 NZ_CP012372.1, WY96-3418 NC_009257.1, WY-00W4114 NZ_CP009753.1, FSC200 NC_019551.1, and LVS NC_07880.1, as well as other references for more detailed IS element content (Rohmer et al., 2007; Svensson et al., 2012; Larson et al., 2015).

b Information within the parentheses denotes the year that the respective *F. tularensis* strain was isolated and the location.

c Includes only full-length IS*Ftu1* ORFs, which is the most abundant transposase/insertion sequence (IS) element in the *F. tularensis* genomes.

d Includes both full-length and remnants of all IS elements in the respective *F. tularensis* genome, including IS*Ftu1* (IS630 family), IS*Ftu2* (IS5 family), *ISFtu3* (ISNCY family, ISHpal-IS1016), IS*Ftu4* (IS982 family), IS*Ftu5* (IS4 family), IS*Ftu6* (IS1595 family), and ISSod13 (IS3 family).

The clinically relevant *F. tularensis* subpopulations that cause tularemia throughout the northern hemisphere include subspecies *tularensis* (or type A) and subspecies *holarctica* (or type B) (Larsson et al., 2009). Due to differences in genomic organization and virulence, the type A clade has been further divided into subtype A.I and A.II, with the A.I strains being recognized as some of the most pathogenic bacteria known (Molins et al., 2010; Birdsell et al., 2014). Like all facultative intracellular pathogens, *F. tularensis* must be able to rapidly gauge the nutritional environment and appropriately adapt. Amino acids have been proven to be critical compounds that allow this pathogen to survive and proliferate (Ziveri et al., 2017). These metabolic compounds contribute to a wide variety of roles beyond protein assimilation, including the production of energy, sulfate assimilation, purine production, carbon acquisition, and cell wall synthesis.

The initial studies in the development of medium with defined components that would support moderate growth of the fastidious pathogen *F. tularensis* established that 13 amino acids, along with spermine or spermidine were needed (Traub et al., 1955; Nagle et al., 1960). Chamberlain further optimized this chemically defined medium (CDM) for enhanced *F. tularensis in vitro* growth (Chamberlain, 1965), which is frequently used for controlled and specialized studies. However, most of the information about amino acid metabolism in the hypervirulent A.I prototype strain SCHU S4 has derived from *in silico* genome analyses, which predicted the absence or incomplete synthesis pathways for at least six amino acids, specifically arginine, cysteine, histidine, lysine, methionine, and tyrosine (Larsson et al., 2005; Rohmer et al., 2007; Meibom and Charbit, 2010). Although attenuated type B LVS is predicted to share similar amino acid auxotrophies with the hypervirulent A.I clade, with the exception of tyrosine in which LVS is capable of synthesizing (Meibom and Charbit, 2010), confirmation of amino acid metabolism in the hypervirulent A.I strains such as the prototype SCHU S4 is needed.

The current study describes major disparities between the *F. tularensis* subpopulations in the metabolic enzymes required for arginine catabolism and subsequent polyamine biosynthesis. Polyamines have been determined to contribute to the ability of bacterial pathogens to adapt and survive, and the processes of polyamine synthesis, uptake, and degradation are coordinated to stringently regulate intracellular levels (Shah and Swiatlo, 2008). We report for the first time that the hypervirulent *F. tularensis* A.I strains can synthesize the polyamines agmatine, putrescine, and spermidine *de novo*, unlike the A.II and B clades, and that this inherent trait promotes a faster replication rate.

## Materials and Methods

### Bacterial Strains and Growth Conditions

The *F. tularensis* strains used in this study included hypervirulent A.I strains SCHU S4 and MA00-2987, virulent A.II WY96-3418, and attenuated B strain LVS, and were all obtained from the Biodefense and Emerging Infections (BEI) Resources, which was established by the National Institute of Allergy and Infectious Diseases (NIAID). Select agent *F. tularensis* strains were transferred to the University of Nebraska Medical Center in Omaha following the requirements of the Federal Select Agent Program as outlined in the Animal and Plant Health Inspection Service/Centers for Disease Control and Prevention (CDC) Form 2, Guidance Document for Request to Transfer Select Agents and Toxin. Manipulation of viable culture material was performed by authorized individuals within a biosafety level 3 (BSL-3) laboratory certified for select agent work by the United States Department of Health and Human Services using laboratory biosafety criteria, according to requirements of the Federal Select Agent Program. For each experiment, *F. tularensis* strains were cultured from a master stock onto Remel Chocolate agar plates (Lenexa, KS) and incubated at 37°C with 5% CO_2_ for 2 days before further subculturing or processing. Growth curves of *F. tularensis* were obtained by culturing in brain heart infusion broth (BHI) (Becton, Dickinson and Company, Sparks, MD) or Chamberlain’s CDM (Chamberlain, 1965) at 37°C with shaking in unbaffled flasks or in a Tecan Spark system in which OD_600_ readings were recorded every hour.

### RNA Preparation and RNA-Seq

For the RNA-Seq libraries, *F. tularensis* SCHU S4, MA00-2987, WY96-3418, and LVS were grown in BHI to mid-exponential growth phase in triplicate and in four independent experiments. *Francisella tularensis* cells were then treated with RNAprotect Bacteria Reagent (Invitrogen) to retain high RNA integrity. Next, FastRNA Pro Blue Kit (MP Biomedicals) or TRIzol (Invitrogen) was used to isolate total RNA, as recommended by the manufacturer. RNA was treated with Baseline-ZERO™ DNase (Epicentre Biotechnologies, San Diego, CA, United States) and the absence of residual genomic DNA was confirmed using control reactions without the addition of reverse transcriptase and subsequent PCR amplification with *Francisella*-specific primer, as previously described (Larson et al., 2011). To determine the RNA concentration, a Qubit fluorometer with the Qubit RNA HS Assay Kit (Invitrogen) were used, as described by the manufacturer. The integrity of the isolated RNA was checked by fractionation in an agarose gel containing ethidium bromide and visualization using a UV transilluminator.

The ScriptSeq Complete Kit for bacteria (Epicentre) was used to generate a ribodepleted, stranded transcriptome of coding and noncoding RNA for each of the four *F. tularensis* strains, specifically SCHU S4, MA00-2987, WY96-3418, and LVS. To monitor the ribosomal RNA removal process, RNA samples were assessed on a Fragment Analyzer (Agilent Technologies, Santa Clara, CA, United States). Twenty-four RNA-Seq libraries were prepared for each of the *F. tularensis* strains, totaling 96 RNA-Seq libraries for statistical power. Deep RNA sequencing of the *F. tularensis* libraries was performed by the University of Nebraska Sequencing Core facility using the Illumina NextSeq platform. Multiplexing of 48 samples per group and two groups in total were each ran on a single Mid-output Illumina flow cell, generating approximately 2.5 million 150 bp paired end reads per sample in 300 cycles.

### Bioinformatic Analyses of RNA-Seq Data

*In silico* analysis of the RNA-Seq data was performed using genomic sequences for the aforementioned *F. tularensis* strains deposited in the National Institute of Health (NIH) GenBank database. The *F. tularensis* genome sequences were obtained from NCBI microbial genome database and had the following accession numbers: SCHU S4 (NC_006570.2), MA00-2987 (NZ_CP012372.1), WY96-3418 (NC_009257.1), WY-00W4114 (NZ_CP009753.1), FSC200 (NC_019551.1), and LVS (NC_07880.1).

Both FastQC and Trimmomatic were used for testing and adjusting the quality of the samples reads. FastQC was initially used for each read to provide quality data, similar to the average Phred score. The Phred score or quality (*Q*) score of a base is an integer value representing the estimated probability of an error (*P*) due to an incorrect base. *Q* and *P* were defined as follows:


$$P=10^{-Q/10}$$

$$Q=-10\;\log_{10}\;P$$

Trimmomatic was utilized to remove the low-quality reads obtained by FastQC. Overrepresented sequences and an average Phred score of less than 20 per base (or 1% probability of an incorrect base) indicates low quality sequences. Therefore, the sequences with the Phred score of less than 20 or overrepresented sequences were eliminated from the reads. After the Trimmomatic modifications, reads with lengths of less than 35 bases were removed from the analysis, and only sequences 35 bases in length or longer were retained. To further ensure quality sequences, FastQC was again applied on the trimmed output results. To align the quality reads for each sample, the splice junction mapper TopHat2 was used (Kim et al., 2013). Bowtie 2 was utilized for indexing the genome assembly (Langmead and Salzberg, 2012). Cufflink identified expressed genes and calculated transcript levels in the aligned reads within the TopHat2 generated Sequence Alignment/Map (SAM) formatted files. The measurement of gene expression was determined using fragments per kilobase of exon per million (FPKM) reads, which quantifies the amount of data that matches a given coding sequence per kilobase length for a given gene or coding sequence per million reads analyzed (Croucher and Thomson, 2010; Giannoukos et al., 2012). The expression level of the same gene from the different *F. tularensis* strains were then compared to the normalized transcriptome data.

*F. tularensis* nucleotide and protein sequences of interest were obtained from the GenBank database. The Basic Local Alignment Search Tool (BLAST), which is provided by the NCBI within the National Library of Medicine (NLM), was used to identify regions of similarity between *F. tularensis* nucleotide or protein sequences. Nucleotide and protein alignments were performed using Clustal Omega (Sievers and Higgins, 2014). Metabolic pathways for gene products of interest were assessed using the Kyoto Encyclopedia of Genes and Genomes (KEGG) analysis tool. Note that as the NCBI annotations continue to evolve with intermittent iterations, *speH* may also be referred to as *speD*.

### Reverse Transcription Quantitative Real-Time PCR

For reverse transcription quantitative real-time PCR (RT-qPCR), *F. tularensis* strains were grown in BHI or CDM as specified in triplicate and in three independent experiments. RNA was isolated, DNase treated, and checked for integrity and concentrations, as described above. To confirm the transcriptional expression of the genes of interest, first-strand cDNA was prepared using the SuperScript IV First-Strand Synthesis System and gene-specific primers, as recommended by the manufacturer (Invitrogen). Next, cDNA was amplified with appropriate gene-specific primer pairs. If conventional RT-qPCR was performed, products were evaluated by agarose gel fractionation, ethidium bromide staining, and densitometry, using ImageJ software (NIH). PowerUp SYBR Green Master Mix (Applied Biosystems) was also used with a QuantStudio 3 Real-time PCR System (Applied Biosystem), according to the manufacturer’s recommendations. Relative gene expression was determined by normalization to the internal control *lpnA* transcript and triplicate samples were evaluated in three independent experiments. Primers utilized for PCR and RT-qPCR are shown in Supplementary Table S1.

### DNA Manipulations and Reagents

All oligonucleotide primers used in this study were synthesized by Thermo Fisher Scientific and are listed in Supplementary Table S1. Genomic DNA was isolated using the Gentra Puregene Cell Kit (Qiagen) or cetyl trimethylammonium bromide (CTAB), according to recommended procedures. Plasmid DNA was prepared with Zymoclean Gel DNA Recovery Kit (Zymo Research Corporation, Irvine, CA, United States), as recommended by the manufacturer. DNA restriction enzyme digests, cloning, and electrophoresis were performed according to standard protocols. PCR was performed using Platinum Taq DNA Polymerase High Fidelity enzyme and associated components (Invitrogen). Ligations were performed using a T4 DNA ligase (Roche) or NEBuilder HiFi DNA Assembly Master Mix (New England Biolabs, Ipswich, MA, United States). DNA fragments were purified using either a QIAquick PCR Purification or QIAquick Gel Extraction Kit (Qiagen). DNA concentrations were obtained using a Nanodrop spectrophotometer or Qubit fluorometer (Invitrogen). DNA sequencing was performed by the University of Nebraska Medical Center Genomics Core Facility using Sanger sequencing. Electroporation of plasmid DNA into *F. tularensis* or *Escherichia coli* was conducted as previously described (Horzempa et al., 2010), and an ECM 630 BTX Electroporation System (BTX Harvard Apparatus) was utilized for these procedures.

### Construction of *Francisella tularensis speE* Deletion Mutants and Expression Plasmids

In-frame and markerless *speE* deletion mutants were generated in *F. tularensis* LVS and SCHU S4 using previously described procedures (Horzempa et al., 2010). To preserve operon integrity and any potential regulatory sites within the target gene sequence, nine consecutive codons at the beginning and end of *speE* were retained in the construct, along with three consecutive stop codons that were inserted after the last of the nine codons at the 5′ region of this gene. Fragments that include these regions and ~500 bp upstream and downstream of the *speE* coding sequence were amplified by PCR, digested, and ligated into PstI/SmaI digested pJH1. The resulting pJH1/*speH*_*speA* construct was DNA sequenced to confirm content and then transferred into *F. tularensis* LVS and SCHU S4 by tri-parental mating, as described previously (Horzempa et al., 2010). Merodiploid strains were recovered and transformed with pGUTS by electroporation for the expression of I-SceI (Horzempa et al., 2010). Colonies resistant to kanamycin were screened by PCR for deletion of the *speE* gene. To cure the *F. tularensis* Δ*speE* mutant of pGUTS, the strains were passaged several times in tryptic soy broth containing cysteine, diluted, and plated on chocolate agar II plates, to obtain isolated CFUs. Plates were incubated for at least 3 days at 37°C with 5% CO_2_ and colonies that formed were replica plated onto chocolate II agar plates with and without kanamycin. Colonies sensitive to kanamycin were isolated and retested for sensitivity to this antibiotic. Deletion of *speE* in the resulting *F. tularensis* LVS Δ*speE* and SCHU S4 Δ*speE* mutants were confirmed by PCR amplification of the relevant chromosomal locus and subsequent fractionation in an agarose gel and staining for comparison to the associated wild-type strain amplicon derived with the same primer pair. Content of this chromosomal locus in the *F. tularensis* LVS Δ*speE* and SCHU S4 Δ*speE* mutants was verified by DNA sequencing. RT-qPCR was used to confirm that *speE* was deleted in the Δ*speE* mutant and that the adjacent genes were transcribed at levels similar to wild-type transcripts.

To complement the Δ*speE* mutant, PCR amplification of the full-length *speE* gene was performed and the resulting amplicon was digested with NheI and BamHI. This PCR product was then ligated into pFNLTP, which was digested with the same restriction endonucleases. The *groESL* or native *spe* promoter were cloned upstream of *speE* into the KpnI and XhoI site of the pFNLTP plasmid containing *speE*. Both the promoter region and the *speE* gene in the pFNLTP expression plasmid were sequenced to verify content. The resulting pFN/Pr_*groESL*/*speE* and pFN/Pr_*spe*/*speE* expression constructs were then electroporated into the *F. tularensis* Δ*speE* mutant for trans-complementation. Expression of *speE* in the complemented Δ*speE* mutant was confirmed by RT-qPCR for triplicate samples in three independent experiments.

### TMT-Labeled Untargeted Mass Spectrometry

Bicinchoninic acid (BCA) assays were performed to determine the concentration of protein in the lysates obtained from *F. tularensis* SCHU S4, MA00-2987, WY96-3418, and LVS. Next, equivalent amounts of protein from the four different *F. tularensis* strains were reduced with DTT, alkylated with iodoacetamide, and digested overnight with sequencing-grade trypsin (Promega). Tryptic peptides were labeled using the Tandem Mass Tag (TMT) 6-plex Reagents (Thermo Fisher Scientific), pooled, and concentrated to 20 μl by vacuum centrifugation. The TMT-labeled peptides were then analyzed using a high-resolution nano-liquid chromatography with tandem mass spectrometry (LC–MS/MS) Tribrid system that included an Orbitrap Fusion with a Lumos coupled to an UltiMate 3,000 HPLC system (Thermo Fisher Scientific). Peptides (500 ng) were run on a pre-column (Acclaim PepMap 100, 75 μm × 2 cm, nanoViper, Thermo Fisher Scientific) and then on an analytical column (Acclaim PepMap RSCL, 75 μm × 50 cm, nanoViper, Thermo Fisher Scientific). The samples were eluted using a 90-min linear gradient of acetonitrile (4%–99%) in 0.1% formic acid.

All LC–MS/MS samples were analyzed using the Protein Discoverer software, version 2.1 (Thermo Fisher Scientific). Sequest HT was set up to search the Swiss-Prot database for both reviewed and unreviewed *F. tularensis* entries. The parameters for Sequest HT were set as follows: enzyme was trypsin, maximum missed cleavage was 2, precursor mass tolerance was 10 ppm, peptide tolerance was ±0.6 Da, fixed modifications were carbamidomethyl (C) and TMT sixplex (any N-terminus), and dynamic modifications was oxidation (M) and TMT sixplex (K). The parameters for Reporter Ion Quantifier were set as follows: integration tolerance was 20 ppm, integration method was most confident centroid, mass analyzer was FTMS, MS order was MS3, activation type was HCD, minimum collision energy was 0, and maximum collision energy was 1,000. The parameters for Percolator were set as follows: target FDR (strict) was 0.01, target FDR (relaxed) was 0.05, and validation was based on *q*-value.

The Proteome Discoverer software version 2.1 (Thermo Fisher Scientific) was again used to normalize the total peptide count for each sample. The algorithm summarizes the peptide group abundance for each sample and determines the maximum sum for all files to obtain a normalization factor. After normalization, the Reporter Ion Quantifier node performs scaling so that the average of all channels is obtained, and the node scales the abundance values of each sample so that the average of all control samples is 100. All other samples are then scaled up or down relative to 100 and when using multiplexed files, the node processes the samples from each file separately. Protein samples were prepared for each of the four *F. tularensis* strains in two independent experiments.

### Statistical Analysis

To determine if differential gene expression was significant, three statistical tests were applied amongst the four *F. tularensis* strains, specifically the two-sample T-test, the Mann–Whitney U-test, and the two-sample Kolmogorov–Smirnov test. The 24 sets of RNA-seq data for each gene in each strain was compared pairwise to each of the other RNA-Seq sets for the same gene in other strains, using the above three statistical tests. A differentially expressed gene was defined as significant if the value of *p* value of all statistical tests for all the pairwise comparisons was less than 0.01 (value of *p* < 0.01).

For RT-qPCR analyses, two-way ANOVA followed by Tukey’s *post hoc* tests were used for statistical analysis when multiple groups were analyzed. Data are presented as means ± SEM and are representative of triplicates in not less than three independent experiments. GraphPad Prism was used for the statistical analyses.

## Results

### Differential Expression of *speHEA* and *aguAB* in *Francisella tularensis* Subpopulations

**Figure 1:**
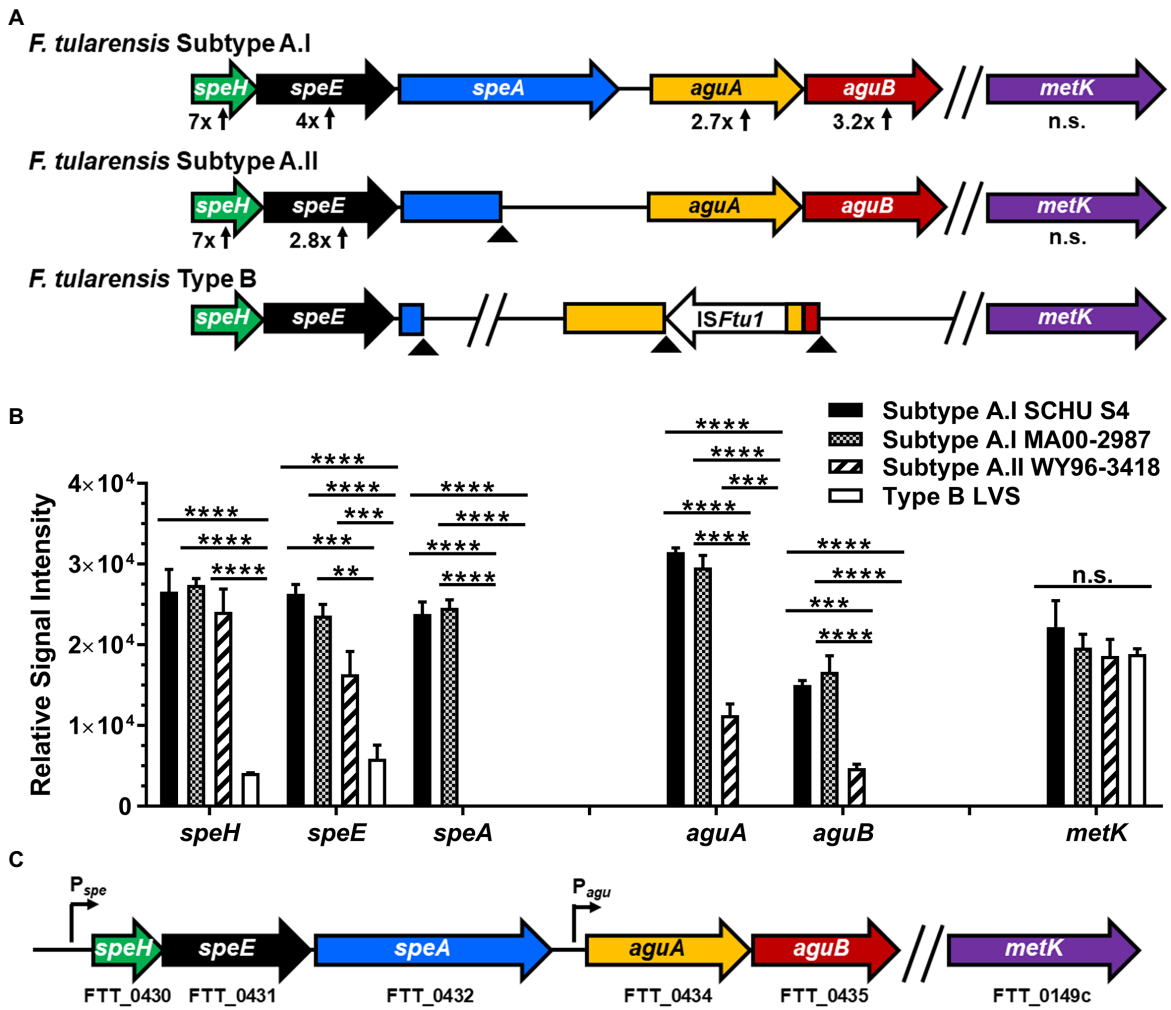
Genetic organization of genes involved in arginine and methionine catabolism and subsequent polyamine biosynthesis within the different *F. tularensis* subpopulations, along with associated transcript levels. **(A)** Diagram depicting the *speHEA* and *aguAB* operons and *metK* gene in the genomes of *F. tularensis* subtype A.I, subtype A.II, and type B clades. **(B)** RT-qPCR results confirming differential expression of the genes within the *speHEA* and *aguAB* operons and *metK* transcript levels in A.I strains SCHU S4 and MA00-2987, A.II strain WY96-3418, and type B strain LVS, during mid-log growth in brain heart infusion broth (BHI). **(C)** Diagram showing the locus tags for prototype A.I strain SCHU S4 associated with *speH*, *speE*, *speA*, *aguA*, *aguB*, and *metK*, as well as the *speHEA* operon promoter (P*_spe_*) and *aguAB* operon promoter (P*_agu_*). In panel **A**, the fold-change increase in mRNA abundance obtained in RT-qPCR relative to the transcript from intact genes in LVS or alternatively WY96-3418 as appropriate, are shown below the associated gene. Arrowheads denote a premature stop codon that results in a truncated gene. In panel **B**, transcript levels obtained in RT-qPCR were normalization to *lpnA* mRNA and the data shown are cumulative of three independent experiments, each conducted with three technical replicates, and are expressed as the mean ± SEM. Data were analyzed using two-way ANOVA with multiple comparisons and Tukey’s *post hoc* tests. Value of *p* > 0.05 were considered not significant (n.s.), and in panel **B**, comparisons that were n.s. are only shown for *metK*. **p < 0.01; ***p < 0.001; and ****p < 0.0001.

To characterize potential differences in the expression of metabolic enzymes within the *F. tularensis* subpopulations, deep RNA sequencing was performed for all three clades. RNA-Seq was performed on representative select agent *F. tularensis* strains that included hypervirulent subtype A.I strains SCHU S4 (A.I prototype) and MA00-2987 and virulent subtype A.II strain WY96-3418 (A.II prototype). Attenuated type B LVS was also evaluated since this non-select agent strain is often studied as a surrogate for this species and may serve as a reference. These strains were cultivated to mid-log in BHI, since growth in this medium recapitulates gene expression by this intracellular pathogen during a macrophage infection (Hazlett et al., 2008). Analysis of the 24 RNA-Seq libraries for each of the four *F. tularensis* strains revealed that most of the genes within the *speHEA* and *aguAB* operons were transcribed at higher levels in the hypervirulent A.I strains compared to subtype A.II WY96-3418 and attenuated type B LVS (Figure 1A). Examination of the genomic organization and content of *speHEA* and *aguAB* in the *F. tularensis* A.I, A.II, and B clades revealed that *speHEA* and *aguAB* were encoded in two adjacent operons (Figure 1A; Supplementary Figure S1). Further, only the A.I genomes contained intact *speHEA* and *aguAB* operons in which all the genes were full-length (Figure 1A; Supplementary Figure S1). More specifically, in the A.II and B genomes, *speA* was truncated. In type B strains, *aguA* and *aguB* were not full-length genes; *aguA* was disrupted by the insertion sequence element IS*Ftu1* and *aguB* had a premature stop codon (Figure 1A; Supplementary Figure S1). Together these analyses revealed that the genomic content of *speHEA*/*aguAB* differed between the *F. tularensis* A.I, A.II, and B clades, but was conserved within each subpopulation, and that only the A.I strains contained all intact genes within these operons.

To verify that the *speHEA* and *aguAB* genes were differentially transcribed in the A.I strains SCHU S4 and MA00-2987, A.II WY96-3418, and type B LVS, as was initially shown in the deep RNA sequencing data, RT-qPCR was performed. These results showed that the *F. tularensis speH* transcript, which is the first gene in the *spe* operon was expressed at a 7-fold higher level in the *F. tularensis* A.I and A.II strains relative to this gene in type B LVS (Figures 1A,B). The adjacent *speE* gene was expressed at a 4- and 2.8-fold higher level in the *F. tularensis* A.I and A.II strains, respectively, compared to type B LVS. The transcripts for *aguA* and *aguB* were 2.7- and 3.2-fold higher in abundance in the A.I strains, respectively, compared to the A.II strain, whereas both *aguA* and *aguB* are disrupted in all type B strains including LVS. Collectively, these data confirmed the differential expression of *F. tularensis speHEA* and *aguAB* and a variable trait between the A.I, A.II, and B subpopulations.

In the *F. tularensis* A.I strains, the stop codon and start codons for *speH* and *speE* overlap by a single bp, *speE* and *speA* are separated by 25 bp, and *aguA* and *aguB* are separated by a single bp, whereas 218 bp separate *speA* and *aguA*. Canonical −35 and −10 promoter elements were only identified upstream of *speH* and *aguA*. More importantly, our RT-qPCR results confirmed that *speHEA* and *aguAB* are two separate operons, each with a separate promoter (Figure 1C).

### Arginine and Methionine Catabolic Pathways

**Figure 2:**
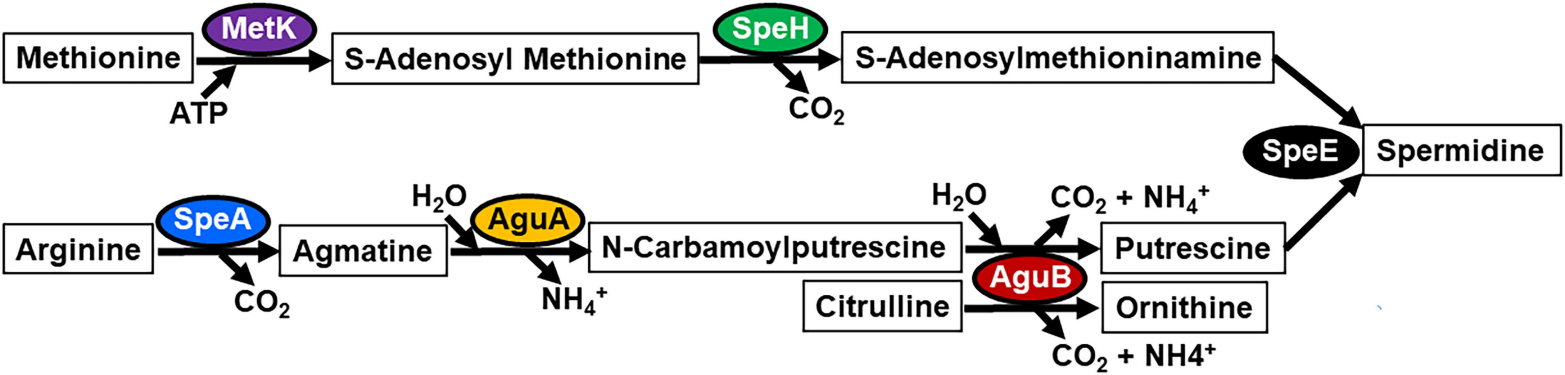
Metabolic pathways for methionine and arginine catabolism and subsequent polyamine biosynthesis in hypervirulent *F. tularensis* A.I strains. Full-length metabolic enzymes in the *F. tularensis* A.I clade include S-adenosylmethionine synthetase (MetK), S-adenosylmethionine decarboxylase (SpeH), arginine decarboxylase (SpeA), agmatine deiminase (AguA), N-carbamoylputrescine amidase (AguB), and spermidine synthase (SpeE).

An examination of the predicted function of *F. tularensis speHEA* and *aguAB* revealed that these genes, along with *metK* that is located at a different chromosomal location, encode enzymes involved in methionine and arginine catabolism and subsequent polyamine biosynthesis (Figure 2). However, unlike the *speHEA* and *aguAB* genes, *metK* transcript levels were similar and only slightly higher in the *F. tularensis* A.I SCHU S4 and MA00-2987strains relative to A.II WY96-3418 and type B LVS (Figure 1B). The genes within the *speHEA* and *aguAB* operons, along with *metK* in the A.I strains encode enzymes that contribute to the catabolism of arginine and methionine and the subsequent biosynthesis of the polyamines agmatine, putrescine, and spermidine (Figure 2). The *metK* gene product S-adenosylmethionine synthetase and ATP are required for the first step in converting methionine to S-adenosylmethionine (SAM; Figure 2). SAM is the principal methyl donor in transmethylation and an aminopropyl donor in polyamine synthesis (Chiang et al., 1996). The next metabolic step for the conversion of SAM to S-adenosylmethioninamine in *F. tularensis* requires the enzyme S-adenosylmethionine decarboxylase, which is encoded by *speH* (Figure 2). This process releases carbon dioxide while producing S-adenosylmethioninamine, one of two substrates required by spermidine synthase SpeE for spermidine biosynthesis in *F. tularensis* (Figure 2). The methionine catabolic enzymes MetK, SpeH, and SpeE are all full-length in the *F. tularensis* A.I, A.II, and B clades, in contrast to the arginine catabolic enzymes SpeA in both the A.II and B subpopulations and AguA and AguB in the type B clade (Figures 1, 2).

In *F. tularensis*, arginine decarboxylase SpeA is responsible for the catabolism of arginine to agmatine and is only full-length in the A.I strains due to premature stop codons within this gene in the A.II and B clades (Figures 1, 2). This catabolic step releases carbon dioxide while producing the substrate agmatine. Next, agmatine deiminase AguA catabolizes agmatine to produce N-carbamoylputrescine in both the A.I and A.II strains, but not the type B strains since this gene is disrupted by the IS element IS*Ftu1* (Figures 1, 2). N-carbamoylputrescine amidase AguB in the *F. tularensis* type A strains has dual enzymatic activity that can catabolizes both N-carbamoylputrescine and citrulline, producing putrescine and ornithine, respectively (Figure 2). The catabolism of these two substrates by AguB results in the production of carbon dioxide and ammonium in both metabolic reactions.

A comparison of the primary sequence for these arginine and methionine metabolic enzymes in the *F. tularensis* A.II and B clades relative to the associated enzyme in the A.I subpopulation was next assessed. SpeH had one conserved residue difference in which the A.I and B strains had a methionine at residue 19 and the A.II strains had a valine at this position (M19V). SpeE had two conserved residues (I186L in both A.II and B strains and K266R in A.II strains), one non-conserved amino acid in A.II strains (G189D), and one non-conserved residue in the type B clade (S107F), relative to this enzyme in A.I subpopulation. Since SpeA was only produced by the A.I clade and not the A.II and B subpopulations, no comparisons could be made for this enzyme. A comparison of AguA and AguB in the A.I and A.II strains revealed that AguA in A.II strains had three non-conservative residue differences (K62N, R199H, and Q253K), whereas AguB was identical in the two type A subpopulations. Relative to S-adenosylmethionine synthetase MetK in the A.I strains, A.II strains had two non-conserved amino acids (E67K and G98D) and type B strains had one non-conserved residue (T105A). Together these results indicated that S-adenosylmethionine decarboxylase SpeH was the most conserved enzyme in all three *F. tularensis* subpopulations in these comparisons, suggesting an important role in SAM catabolism. SpeH also provides one of the two substrates required for spermidine biosynthesis in the A.I strains (Figure 2), indicating an additional vital role within this species.

### Chromosomal Location of *Francisella tularensis spe*/*agu* Operons and *metK*

**Figure 3:**
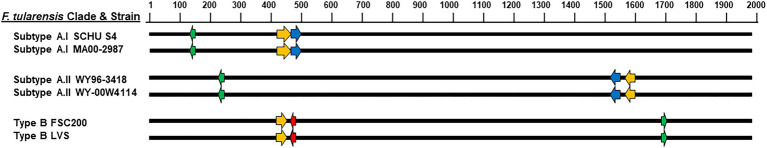
Diagram depicting chromosomal location and directionality of the *speHEA*/*aguAB* operons and the *metK* gene in representative *F. tularensis* subtype A.I, subtype A.II, and type B strains. The relative nucleotide position in kilobase pairs of the double-stranded DNA genome in *F. tularensis* is shown at the top, and the circular chromosome in *F. tularensis* was linearized for this figure using *dnaA* as the initial coding sequence after the first base pair. Representatives from the *F. tularensis* subpopulations include subtype A.I strains SCHU S4 and MA00-2987, subtype A.II strains WY96-3418 and WY-00W4114, and type B strains FSC200 and LVS. The *speHEA* and *aguAB* operons are represented by the adjacent orange and blue arrows, respectively, and *metK* is represented with a green arrow. The IS*Ftu1* insertion sequence element in the type B *aguA* gene is denoted with a red arrow. The arrows show the direction of the operon and gene coding sequences and were enlarged for visualization. Nucleotide sequences in the NCBI database were used to position the operons and genes of interest in the *F. tularensis* genomes, and the associated accession numbers are described in the Materials and Methods section.

The chromosomal location and directionality of the *speHEA*/*aguAB* operons and *metK* were compared in the *F. tularensis* A.I, A.II, and B strains. These results revealed that the spatial location and directionality of the *spe*/*agu* operons differed the most between the A.I and A.II strains and were the most similar in the A.I and B clades, even though only *speH* and *speE* are intact in the type B strains (Figure 3). In contrast, *metK* in the A.I and A.II clades was positioned in a similar chromosomal location and direction, and the type B *metK* differed in both aspects relative to the type A strains. However, in all three *F. tularensis* subpopulations, *metK* was positioned near the gene encoding the bacterial replication initiator protein DnaA, with the A.I *metK* being the closest to *dnaA* (Figure 3). Although all *F. tularensis* subpopulations share numerous features and have numerous IS elements, the type B strains have considerably more IS elements, which has ostensibly contributed to the higher abundance of pseudogenes in this clade (Table 1). Moreover, the most abundant IS element, specifically IS*Ftu1* from the IS630 family of transposases was responsible for the disruption of *aguA* in the type B clade.

The genes adjacent to the *speHEA*/*aguAB* operons in the *F. tularensis* A.I, A.II, and B clades were conserved in organization and directionality, despite the genomic, transcriptomic, and proteomic differences within the *spe*/*agu* operons. In all three subpopulations, threonine synthase *thrC1* was adjacent and 5′ to *speH*, while UDP-2,3-diacylglucosamine hydrolase *lpxH* was adjacent and 3′ to the *agu* operon (Table 2; Supplementary Figure S2A). The proteins encoding by *thrC1* and *lpxH* both provide critical cellular functions; ThrC1 catalyzes the production of threonine and LpxH is involved in lipid A biosynthesis. The *thrC1* gene was transcribed in the same direction as the *speHEA*/*aguAB* operons, whereas *lpxH* was expressed in the opposite orientation. A remnant of a beta-galactosidase gene encoding only the first 76 amino acids of the 656-residue full-length enzyme also separated *thrC1* and *speH* and was transcribed in the opposite direction of these two genes. This beta-galactosidase gene remnant shared the highest homology to associated enzyme in *Burkholderia* and *Neisseria*.

**Table 2:** Genes adjacent to *speHEA*/*aguAB* operons and *metK* in *F. tularensis* subpopulations.^a^

Clade	Gene(s)^a^	5′ Adjacent gene	3′ Adjacent gene
Subtype A.I	*speHEA*/*aguAB*	Threonine synthase *thrC*	UDP-2,3-diacylglucosamine hydrolase *lpxH*
Subtype A.I	*metK*	Fatty acid desaturase	30S Ribosomal protein S16 *rpsP*
Subtype A.II	*speHEA*/*aguAB*	Threonine synthase *thrC*	UDP-2,3-diacylglucosamine hydrolase *lpxH*
Subtype A.II	*metK*	Fatty acid desaturase	30S Ribosomal protein S16 *rpsP*
Type B	*speHEA*/*aguAB*	Threonine synthase *thrC*	UDP-2,3-diacylglucosamine hydrolase *lpxH*
Type B	*metK*	Fatty acid desaturase	30S Ribosomal protein S16 *rpsP*

a Supplementary Figure S2 shows a diagram with the directionality of the 5′ *and 3*′ adjacent genes to the *speHEA*/*aguAB* operons and *metK* in *F. tularensis*.

The genes flanking *metK* were also highly conserved within the *F. tularensis* A.I, A.II, and B subpopulations. A gene encoding a fatty acid desaturase was located 5′ to *metK*, but in the opposite orientation, while *rpsP* was positioned 3′ to *metK* and also oriented in the opposing direction, indicating that *metK* is monocistronic (Table 2; Supplementary Figure S2B). The *rpsP* gene encodes the 30S ribosomal protein S16 that is highly expressed and required for protein translation. The conservation of the genes and associated regulatory regions adjacent to the *speHEA*/*aguAB* operons and *metK* gene in the different *F. tularensis* subpopulations may provide a fitness advantage to this facultative intracellular pathogen.

### Transcriptional Expression of *Francisella tularensis speHEA*, *aguAB*, and *metK*

The *F. tularensis* promoter region for the *speHEA* operon was 586 bp in length in the A.I and A.II clades and 564 bp in the B strains due to a 22 bp deletion. The same 22 bp sequence was located directly after the threonine synthase *thrC* coding sequence in the type A strains, whereas in the type B clade this sequence was incorporated into the 3′ end of the slightly shorter *thrC* open reading frame. The type B strains also had a single adenine insertion and a SNP that was a thymine instead of a guanine compared to the type A strains in the *spe* promoter region. A putative OmpR-like response regulator binding sequence (5′-TTGTAGCA-3′) was located between the −35 and −10 region in the *spe* promoter and was conserved within the A.I, A.II, and B clades. Although type A strains encode two OmpR-like orphan response regulators that are 228 and 229 residues in length with 54% amino acid similarity, type B strains only encode one of these regulatory factors. These and other differences in regulatory capabilities between these subpopulations may have contributed to the reduced expression levels of *speH* and *speE* in type B LVS relative to the A.I and A.II strains (Figure 1B).

A comparison of the nucleotide content in the 218 bp region separating the *spe* and *agu* operons in the A.I, A.II, and B clades revealed four SNPs. These SNPs included three guanines and one thymine in subtype A.I; two guanines and two thymines in subtype A.II; and one guanine, two adenines, and one thymine in type B strains. One SNP comprised the last 3′ nucleotide in the Pribnow box/−10 region, one SNP followed the Shine-Dalgarno/ribosomal binding site, and the remaining SNPs were in the intergenic region between the *spe* and *agu* operons. Overall, the A.II and B clades have more A/T enrichment in the intergenic nucleotides between the *spe* and *agu* operons than the A.I strains.

The *F. tularensis metK* promoter region was 293 bp and highly conserved in the A.I, A.II, and B subpopulations with only two SNPs, neither of which were located in the predicted Pribnow box/−10 region, −35 region, nor Shine-Dalgarno/ribosomal binding site. One SNP was a thymine in the A.I and B clades and a cytosine in the A.II strains. The other SNP was an adenine in the A.I clade, whereas the A.II and B strains had a guanine in this position. Interestingly, the promoter for *metK* and the adjacent *rpsP* gene, which encodes the 16S ribosomal protein, share the same predicted Pribnow box/−10 region that was located approximately in the middle of this promoter region. In addition to *rpsP*, other essential genes that are highly expressed during bacterial replication were located nearby to *metK*, including genes that encode 50S ribosomal proteins, tRNAs, and DNA-directed RNA polymerase subunits. These analyses support the premise that *metK* and *rpsP* are co-regulated and transcribed during favorable growth conditions for rapid replication.

A global RNA-Seq analysis of the immortalized mouse monocyte cell line P388D1 infected with *F. tularensis* LVS reported a 6.2- and 4.8-fold increase in *speH* and *speE* at 4 h post infection (hpi), respectively, and then a decrease in their abundance by 8 hpi (Bent et al., 2013). These results suggest that *speH* and *speE* contribute to the phagosomal escape of this intracellular pathogen, even though *speA*, *aguA*, and *aguB* are not intact in type B strains. Another notable global transcriptome study showed that the expression of the polyamine synthesis genes *speHEA*, *aguAB*, and *metK* were upregulated in primary mouse macrophages infected with *F. tularensis* SCHU S4 (Wehrly et al., 2009). Collectively, these data demonstrate that these metabolic gene products provide an important role in the persistence of *F. tularensis*, particularly for hypervirulent A.I SCHU S4.

### Protein Expression of *Francisella tularensis speHEA*, *agu*, and *metK*

Untargeted proteomic data from subtype A.I strains SCHU S4 and MA00-2987, subtype A.II strain WY96-3418, and type B LVS grown in BHI to mid-log growth phase were obtained using TMT mass spectrometry. These results revealed that AguB was 3.7- and 4-fold more abundant in the A.I strains SCHU S4 and MA00-2987, respectively, relative to this enzyme in A.II WY96-3418. MetK levels were similar in the A.I strains SCHU S4 and MA00-2987 and were only 1.7- and 1.8-fold higher when compared to the abundance of this enzyme in the A.II and B strains, respectively. In SCHU S4 and MA00-2987, MetK was 2-fold more abundant than AguB. SpeH, SpeE, SpeA, and AguA, however, were not detected in these analyses.

A comparison of proteins produced by *F. tularensis* LVS grown in several different culture broths showed similar MetK levels, while SpeE abundance was low or undetectable (Holland et al., 2017). In another global proteomic investigation, MetK was detected in both LVS and SCHU S4, whereas AguA, AguB, SpeE were only identified in SCHU S4 with just one of the two biological replicates detecting SpeE (Lenco et al., 2009). Collectively, these results indicated that although all the *F. tularensis* clades can metabolize methionine to produce SAM and S-adenosylmethioninamine, only the hypervirulent A.I strains can produce the polyamines agmatine, putrescine, and spermidine *de novo via* the enzymes encoded by *speHEA*, *aguAB*, and *metK*.

### Construction and Characterization of *Francisella tularensis* Δ*speE* Mutants

**Figure 4:**
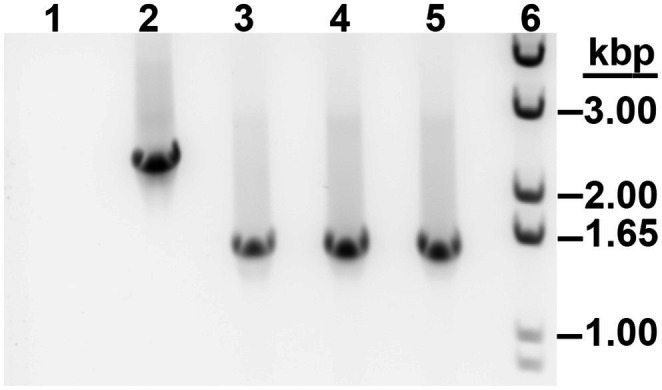
PCR amplification of the *speHEA* chromosomal locus in *F. tularensis* A.I SCHU S4 wildtype and the isogenic Δ*speE* mutants. Shown are SCHU S4 wildtype (lane 2) and the isogenic Δ*speE* mutant used for this study (lane 3), along with two additional Δ*speE* mutants (lanes 4 and 5). The no template control (lane 1) and dsDNA marker (lane 6) are also shown.

To determine the contribution of spermidine synthase SpeE in *F. tularensis*, chromosomal *speE* deletion mutants were generated in hypervirulent A.I SCHU S4 and type B LVS, since this enzyme was intact in both clades. The methods used to generate markerless and in-frame *speE* deletion mutants (Δ*speE*) in *F. tularensis* were carried out as previously described (Horzempa et al., 2010). PCR amplification of the *speHEA* chromosomal locus in the *F. tularensis* SCHU S4 Δ*speE* and LVS Δ*speE* mutants confirmed that the 0.8 kbp *speE* gene was deleted from the genome of the respective wild-type strain (Figure 4; Supplementary Figure S3). DNA sequencing of this region confirmed the expected content and verified that the adjacent genes *speH* and *speA* were not disrupted. RT-qPCR validated that the absence of *speE* mRNA in the *F. tularensis* Δ*speE* mutants and that no polar effects occurred in the transcription of the adjacent upstream and downstream genes in the SCHU S4 Δ*speE* mutant relative to wild-type SCHU S4 (Supplementary Figure S4).

**Figure 5:**
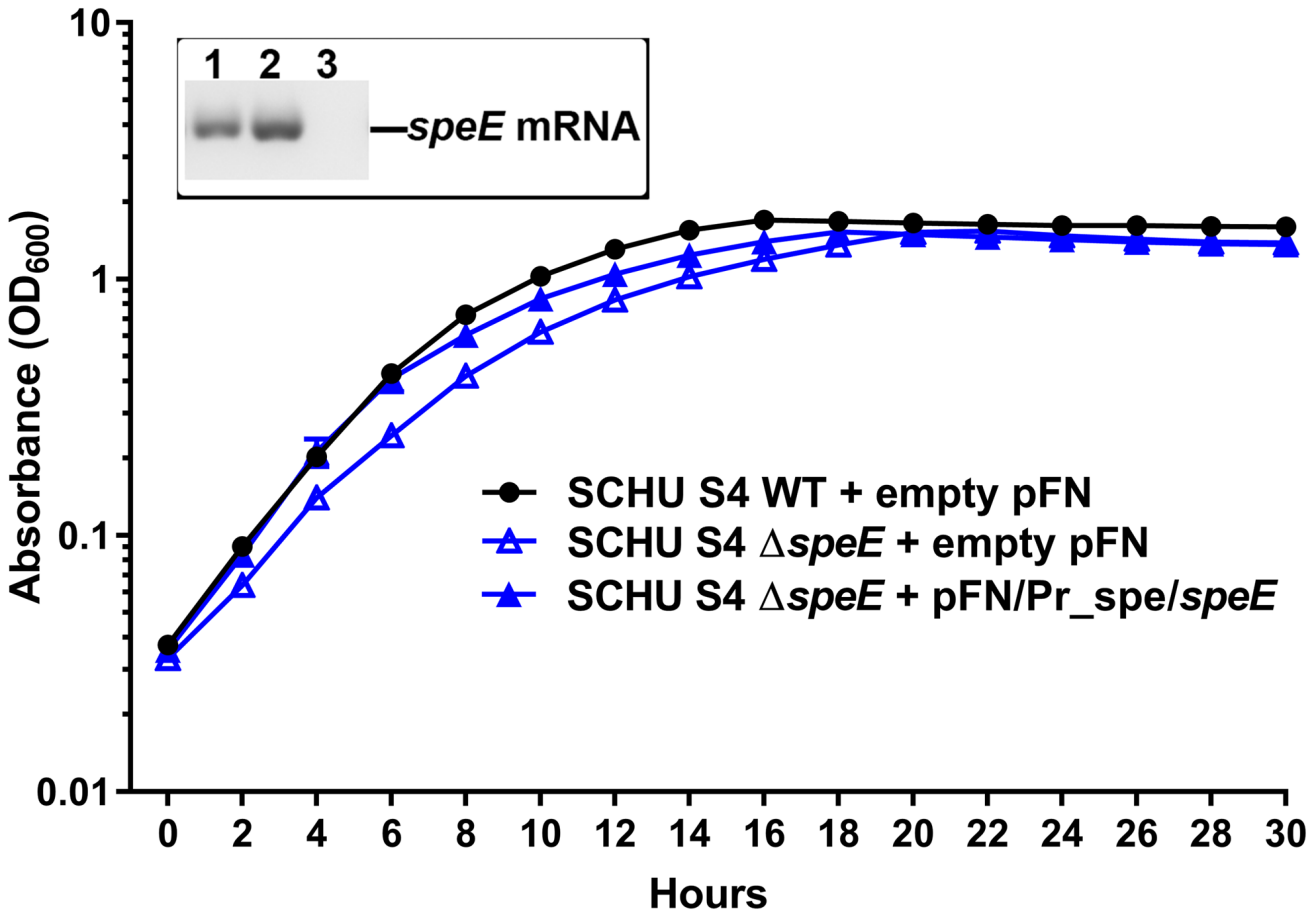
Trans-complementation of the *F. tularensis* SCHU S4 Δ*speE* mutant. Growth of wild-type SCHU S4 and the isogenic SCHU S4 Δ*speE* mutant that contained an empty pFN plasmid was compared to the in *trans* complemented SCHU S4 Δ*speE* mutant containing the *speE* expression plasmid pFN/Prˍ*spe*/*speE* during growth in Chamberlain’s chemically defined medium (CDM). Insert shows representative RT-qPCR products obtained with *speE*-specific primers for the presence or absence of *speE* transcripts as appropriate for wild-type SCHU S4 containing empty plasmid (lane 1), complemented SCHU S4 Δ*speE* mutant containing expression plasmid pFN/Prˍ*spe*/*speE* (lane 2), and SCHU S4 Δ*speE* mutant containing empty plasmid (lane 3). The mean with ± SEM is shown for the growth curves obtained from triplicate samples in three independent experiments.

To assess if the deletion of *speE* altered growth, a comparison of wild-type SCHU S4 and wild-type LVS to the associated and isogenic Δ*speE* mutant was evaluated. These results showed that SCHU S4 Δ*speE* grew slower than wild-type SCHU S4, whereas the LVS Δ*speE* mutant grew faster than wild-type LVS in Chamberlain’s CDM, suggesting that spermidine synthase SpeE provides a fitness advantage to SCHU S4 and not LVS. To ensure that no polar and off-target effects occurred during the process of producing the SCHU S4 Δ*speE* mutant that contributed to this phenotype, trans-complementation was performed. For these assessments, the SCHU S4 Δ*speE* mutant was complemented in *trans* with the *Francisella* expression plasmid pFNLTP containing the constitutive *groESL* promoter upstream of *speE* (pFN/Prˍ*gro*/*speE*). However, these complementation experiments were unsuccessful, so the native *spe* promoter was then utilized to regulate *speE* expression. The usage of the native *spe* promoter in pFNLTP (pFN/Prˍ*spe*/*speE*) successfully complemented the SCHU S4 Δ*speE* mutant in *trans*, resulting in growth comparable to wild-type SCHU S4 (Figure 5). As shown in the Figure 5 insert, *speE* transcripts were present in wild-type SCHU S4 with empty plasmid and the complemented SCHU S4Δ*speE* mutant containing the pFN/Prˍ*spe*/*speE* expression plasmid, but not the SCHU S4Δ*speE* mutant with empty plasmid. These data validated that *speE* is not transcribed by *F. tularensis* SCHU S4 Δ*speE* and that the reduced growth rate of this mutant can be complemented.

### Transcriptional Regulation of *speHEA*, *aguAB*, and *metK* in Wildtype and Δ*speE* Mutant

To determine if arginine and methionine concentrations contributed to the regulation of the transcriptional expression of the *speHEA*/*aguAB* operons and *metK* by *F. tularensis* SCHU S4 and the isogenic Δ*speE* mutant, RT-qPCR was performed. For these experiments, the *F. tularensis* strains were cultured in modified CDM containing 0, 0.3, 3, or 6 mM of arginine or methionine. When the bacteria were in mid-log growth phase, RNA was isolated and prepared for RT-qPCR, as described in the Materials and Methods section. No significant change in transcript abundance was observed for the *spe*, *agu*, and *metK* genes (data not shown). Further, an examination of the transcript levels for the proposed arginine and methionine importers ArgP and MetNIQ, respectively, did not change during growth with different concentrations of these essential amino acids (data not shown). These results indicated that the transcriptional expression of *F. tularensis* A.I *speHEA*/*aguAB* operons and *metK*, as well as the amino acid transporters ArgP and MetNIQ is not regulated by just the levels of these essential amino acids.

Next, we sought to determine if stress conditions altered the transcriptional expression of the genes in the polyamine biosynthesis pathway in *F. tularensis* SCHU S4 and the isogenic Δ*speE* mutant when exposed to acidic conditions, hydrogen peroxide, and nitric oxide. After exposure to a low pH of 5.2 in CDM adjusted with hydrochloric acid for 1 h, 1 mM hydrogen peroxide for 1 h, and 100 μM of the nitric oxide-generating compound S-nitroso-N-acetylpenicillamine (SNAP) for 0.5 h, RNA was extracted and evaluated by RT-qPCR. Again, these conditions did not significantly increase nor decrease the transcript levels for these genes (data not shown). Therefore, a combination of stress factors and/or different conditions may be needed to modify the transcriptional expression of the *speHEA*, *aguAB*, and *metK* genes in *F. tularensis* SCHU S4 and the isogenic Δ*speE* mutant. Alternatively, these gene products may be regulated at the translational or post-translational level to promptly respond to internal and external nutritional status and stress.

Charity and associates showed that LVS *speH* transcript levels were 3.3- and 3.9-fold lower in Δ*mglA* and Δ*sspA* deletion mutants, respectively (Charity et al., 2007). Since MglA and SspA facilitate sigma factor RpoD (σ^70^) binding to DNA to regulate virulence and virulence-enhancing genes (Travis et al., 2021), these findings suggest that these transcription factors regulate *spe* operon expression and that these genes may contribute to the virulence of *F. tularensis*. Therefore, our ongoing studies will investigate the various factors that regulate the polyamine biosynthesis genes *speHEA*, *aguAB*, and *metK*, along with the role of the associated metabolic enzymes in *F. tularensis* pathogenicity.

### Growth Comparison of *Francisella tularensis* Wildtype and Δ*speE* Mutant Strains

**Figure 6:**
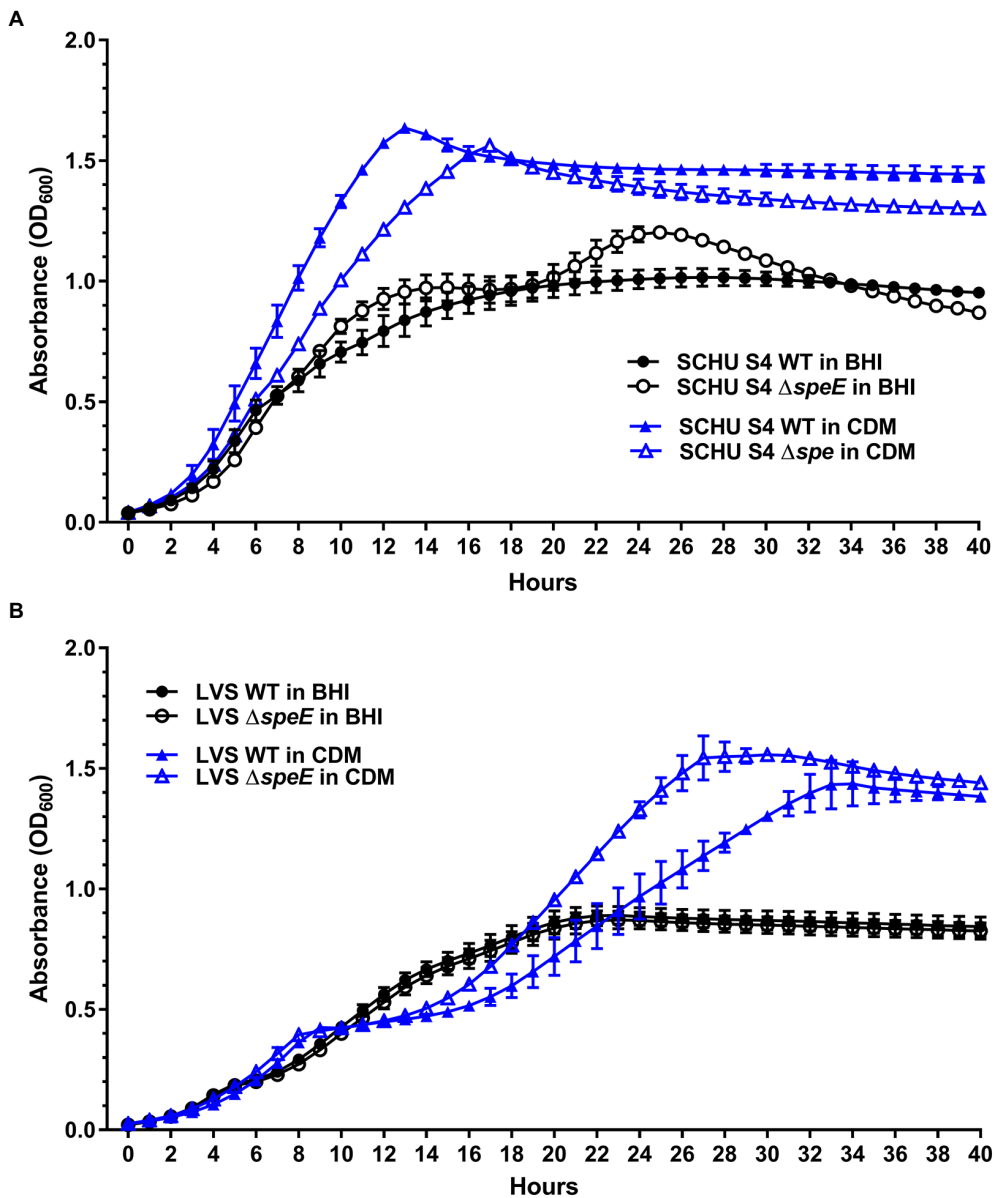
Growth comparison of *F. tularensis* subtype A.I SCHU S4 and type B LVS versus the associated and isogenic Δ*speE* mutant in brain heart infusion broth (BHI) and Chamberlain’s chemically defined medium (CDM). **(A)** *F. tularensis* SCHU S4 wildtype (WT) growth versus the isogenic Δ*speE* mutant and **(B)** *F. tularensis* LVS wildtype (WT) versus the isogenic Δ*speE* mutant in BHI (black circles) and Chamberlain’s CDM (blue arrowheads). WT strains are denoted with filled symbols and the Δ*speE* mutants are indicated with unfilled symbols. The mean with ±SEM for triplicate samples in three independent experiments is shown.

To evaluate the growth rate and yield of *F. tularensis* SCHU S4 and LVS relative to the isogenic Δ*speE* mutants in different media, these strains were grown in Chamberlain’s CDM and in BHI. These assessments demonstrated that all four strains grew to a higher cell density in Chamberlain’s CDM, which contains the polyamine spermidine or spermine (Chamberlain, 1965), in comparison to BHI (Figure 6). These results validated that the components within Chamberlain’s CDM provide the appropriate provisions for more efficient *in vitro* growth by *F. tularensis*, as well as corroborates with the data obtained by others (Mc Gann et al., 2010).

In BHI, *F. tularensis* SCHU S4 and the SCHU S4 Δ*speE* mutant differed in growth rate and maximum cell density (Figure 6A), whereas the growth of wild-type LVS and the LVS Δ*speE* mutant was similar (Figure 6B). Unlike wild-type SCHU S4, the SCHU S4 Δ*speE* mutant exhibited diauxic growth in BHI and grew at a faster rate than wild-type SCHU S4 during these two exponential phases. The SCHU S4 Δ*speE* mutant also reached a slightly higher cell density (maximum OD_600_ of 1.2) than wild-type SCHU S4 (maximum OD_600_ of 1.0), which occurred after 25 h of growth in BHI (Figure 6A). However, after SCHU S4 Δ*speE* reached maximum cell density, cell death occurred with a concomitant and continuous decrease in cell density. Unlike the SCHU S4 Δ*speE* mutant, wild-type SCHU S4 did not grow in a diauxic manner in BHI, but rather exhibited the canonical bacterial lag, exponential growth, and stationary phases. After 26 h of growth in BHI, SCHU S4 cell density reached a maximum OD_600_ of 1.0 and remained mainly unchanged in stationary phase for at least 48 h when the experiments were terminated. Total cell density based on the area under the growth curve for SCHU S4 and SCHU S4 Δ*speE* grown in BHI was 32.13 ± 0.26 and 33.89 ± 0.20, respectively.

For both wild-type LVS and LVS Δ*speE* cultured in BHI, the growth rate slightly decreased after 13 h until maximum cell density was obtained (OD_600_ of 0.8), which occurred after a total of 22 h when the cells were entering stationary phase (Figure 6B). The overall cell density based on the area under the growth curve for LVS and LVS Δ*speE* grown in BHI was 26.02 ± 0.22 and 25.28 ± 0.20, respectively. Since growth rate and cell density were similar between wild-type LVS and the isogenic Δ*speE* mutant, these results indicated that SpeE spermidine synthase does not substantially contribute to the fitness of these type B strains when cultured in BHI. In contrast, these findings indicated that spermidine synthase SpeE promotes steady state growth in SCHU S4 and implicates that spermidine production by this enzyme may also provide a protective function against factors that promote cell death during growth in BHI.

In Chamberlain’s CDM, both *F. tularensis* SCHU S4 and SCHU S4 Δ*speE* grew substantially faster than LVS and LVS Δ*speE*, with wild-type SCHU S4 growing 3.8-fold faster than wild-type LVS. The generation time of 1.22 h was obtained for SCHU S4, whereas LVS had a doubling time of 4.66 h (Figure 6B). However, wild-type LVS and the LVS Δ*speE* mutant eventually reached a similar maximum cell density after 33 h (OD_600_ of 1.4) and 27 h (OD_600_ of 1.53), respectively (Figure 6B). In comparison, wild-type SCHU S4 and the SCHU S4 Δ*speE* mutant grown in CDM reached the highest cell density after 12 h (OD_600_ of 1.6) and 17 h (OD_600_ of 1.54), respectively (Figure 6A). Further, in CDM both LVS and the LVS Δ*speE* mutant showed diauxic growth (Figure 6B), which was not the case for SCHU S4 and the SCHU S4 Δ*speE* mutant (Figure 6A). In CDM, the total cell density based on the area under the growth curves for SCHU S4 and SCHU S4 Δ*speE* was 50.07 ± 0.20 and 44.98 ± 0.12, respectively. For LVS and LVS Δ*speE* grown in CDM, the overall cell density based on the area under the growth curves was 31.29 ± 0.33 and 36.61 ± 0.17, respectively. In general, these results demonstrated that spermidine synthase SpeE in wild-type SCHU S4 contributes to an enhanced growth rate and cell yield in CDM, whereas the deletion of *speE* in LVS augmented the growth rate and slightly increased cell yield in CDM (Figure 6).

## Discussion

In the current study, we showed that considerable differences exist between *F. tularensis* subpopulations in the metabolic pathways associated with arginine and polyamine metabolism, but not methionine catabolism. Hypervirulent subtype A.I strains such as SCHU S4 and MA00-2987 have intact *speHEA* and *aguAB* operons, whereas subtype A.II strains have a prematurely terminated *speA* gene and type B strains have truncated *speA* and *aguAB* genes (Figure 1A; Supplementary Figure S1). Moreover, the *F. tularensis* A.I strains SCHU S4 and MA00-2987 expressed higher levels of all the genes involved in arginine and polyamine metabolism within these two operons. Inactivation of a gene(s) in an operon or pathway will most likely promote an increase in the frequency of A/T mutations and ongoing erosion (Ochman, 2003; Bobay and Ochman, 2017), which appears to be the case within these altered regions in the *F. tularensis* A.II and B clades. These modifications are common in bacteria whose ecology includes an intracellular niche (Balbi et al., 2009). Implication of lineage derivations and genetic polymorphisms has been previously discussed (Svensson et al., 2005; Larson et al., 2015). Nevertheless, the disparities in the *spe*/*agu* arginine and polyamine metabolic pathways within the *F. tularensis* subpopulations suggests that the A.II and B clades must utilize alternative and available mechanisms to acquire essential polyamines.

The initial enzyme involved in the *spe*/*agu* arginine catabolism and polyamine biosynthesis pathway is arginine decarboxylase SpeA, which was only intact in *F. tularensis* A.I strains. Since *F. tularensis* does not have the biosynthetic pathways to synthesize arginine, this essential amino acid must be imported for numerous cellular functions such as protein production, as well as *de novo* polyamine biosynthesis in the A.I clade. The genome of this intracellular pathogen does not encode a protein with homology to an ArgR-like repressor for the regulation of arginine metabolism, but there may be other unidentified regulators. Further, since arginine abundance and the stress factors examined did not substantially alter the transcriptional expression of the *spe*/*agu* genes nor *argP*, which encodes an arginine importer, regulation may be occurring post-transcriptionally and/or post-translationally. Alternatively, additional factors are required or *F. tularensis* SCHU S4 may endogenously have the capability to import this critical amino acid or peptides containing this residue in a steady state manner.

In addition to arginine catabolism by arginine decarboxylase SpeA, there are three additional enzymes encoded in the *F. tularensis* A.I genome that can hydrolyze arginine. These enzymes are annotated in the National Center for Biotechnology Information (NCBI) database as arginine-like deiminases and catalyze the conversion of arginine to the non-proteinogenic amino acid citrulline, as well as ammonium. The importance of arginine deiminases in a bacterial species with a highly reduced genome was shown to facilitate adaptation from the environment to a mammalian niche, due to the ability of these enzymes to confer protection against acid stress through ammonia production (Tian et al., 2022). In *F. tularensis* A.I genomes, these genes encode a 304-residue and a 307-residue arginine-like deiminase, which have 48% identity in 146 residues and 64% similarity in 195 residues to each other, whereas the third gene encodes a 183-residue protein with no homology to the two larger enzymes. The smallest 183-residue arginine-like deiminase shares the highest homology to a 261-residue amidinotransferase in *Candidatus Roizmanbacteria* (40% identity in 73 out of 183 residues and 62% similarity in 113 out of 183 residues) and another water-associated bacterium, specifically *Methylocystis* (39% identity in 72 out of 187 residues and 58% similarity in 110 out of 187 residues). *Francisella tularensis* A.II genomes contain the 183-residue and 304-residue arginine-like deiminases while the type B genomes encode only the 307-residue arginine-like deiminase, exemplifying more metabolic differences within these subpopulations.

AguB N-carbamoylputrescine amidase, which is only intact in the *F. tularensis* A.I and A.II strains and not the type B clade, hydrolyzes citrulline and N-carbamoylputrescine, producing ornithine and the polyamine putrescine, respectively. Citrullinase activity has been historically used to differentiate *F. tularensis* type A strains from type B strains due to the differentiating capability of AguB in the type A clade to catabolize citrulline to ornithine. An investigation by others evaluated a SCHU S4 Δ*aguB* mutant and reported that no growth defect in culture nor infected macrophages was observed; however, mice infected with this mutant lived longer and had a significantly reduced bacterial burden in the lungs, liver, and spleen relative to wildtype infected mice, indicating that *aguB* contributes to intracellular persistence and pathogenesis (Mahawar et al., 2009). Catabolism by arginine deiminase and N-carbamoylputrescine amidase AguB, as well as agmatine deiminase AguA all generate ammonium, which may contribute to basification of the surrounding environment and/or to a metabolic signaling network for an appropriate cellular response. However, the functional contribution of these enzymes and resulting products to the fitness of *F. tularensis* will require further study.

Methionine serves as an initiation amino acid in protein translation in all domains of life (Kozak, 1999). This essential amino acid can be converted to SAM (Cantoni, 1953), and in *F. tularensis*, SAM is derived from the catabolism of methionine by SAM synthetase MetK (Figure 2). SAM provides an activated methyl group donor for many fundamental cellular processes, including the methylation of proteins and nucleic acids and serves as a substrate for spermidine biosynthesis. This metabolite is the second most used biological compound after ATP (Cantoni, 1975). Analysis of a *F. tularensis* SCHU S4 transposon library revealed that *metK* contributed to fitness and was essential (Ireland et al., 2019). Our results corroborate with these findings and show that the transcriptional and translational expression of *metK* is similar in the *F. tularensis* A.I, A.II, and B clades with only slightly higher levels in the A.I strains. An evaluation of the *metK* promoter in representative strains from each of these subpopulations revealed a highly conserved 293 bp region with only two nucleotide differences. This promoter region also controls the adjacent and highly transcribed gene *rpsP*, which encodes the ribosomal protein S16 in the 30S small subunit of the ribosome that is required to stabilize this complex. The conservation of this promoter region and shared Pribnow/−10 region that regulates both the expression of the SAM synthetase *metK* gene and the divergently transcribed *rpsP* gene implicates the presence of highly regulated *cis*-acting elements that sense metabolite abundance to co-regulate these vital gene products. However, neither low nor high methionine abundance nor the stress conditions examined substantially altered the transcriptional expression of *metK* or the genes that encode the methionine importer MetNIQ in wild-type SCHU S4 or the isogenic Δ*speE* mutant. These data suggest that *F. tularensis metK* expression may be needed to produce SAM even when conditions are not optimum and that the abundance of this transcript is tightly regulated, supporting the critical role of this multifunctional metabolite that includes serving as a substrate for spermidine biosynthesis in the A.I strains.

In the *F. tularensis* A.I clade, the catabolism of arginine produces the polyamines agmatine and diamine putrescine, whereas the metabolism of both arginine and methionine is required to provide the substrates for *de novo* biosynthesis of the triamine spermidine (Figure 2). Polyamines are small organic polycations with amine groups that are found in nearly all living organisms and are involved in a variety of essential biological processes (Igarashi and Kashiwagi, 2000, 2010). Studies have shown that polyamines modulate chromatin organization, DNA replication, transcription, translation, ion transport, and membrane dynamics by the electrostatic interactions of these small polycationic molecules with negatively charged macromolecules, including DNA, RNA, proteins, and phospholipids (Wortham et al., 2007; Shah and Swiatlo, 2008; Michael, 2018). The ability of polyamines to enhance the replication rate of *Francisella* was discovered during the efforts of others to produce a chemically defined medium (Traub et al., 1955; Nagle et al., 1960; Chamberlain, 1965). Moreover, other studies reported on spermine-responsive regions within *F. tularensis* IS elements that regulate the expression of several adjacent genes (Carlson et al., 2009), and identified a spermine-responsive gene that was shown to be involved in virulence (Russo et al., 2011).

The results from the current study comparing *F. tularensis* wild-type A.I and B strains with the isogenic Δ*speE* mutants demonstrated that SpeE spermidine synthase contributes to an increase in the growth rate of subtype A.I SCHU S4 and conversely hinders the growth rate of type B LVS in Chamberlain’s CDM. In BHI, SCHU S4 Δ*speE* grew similar to wildtype, but unlike wildtype, this mutant exhibited diauxic growth and began to lyse during stationary phase. Since BHI has a high content of essential nutrients, including polyamines and peptides as an amino acid source, this medium is often used to cultivate fastidious pathogens. Therefore, although SCHU S4 Δ*speE* can no longer synthesize spermidine *de novo*, BHI provides polyamines, as well as amino acids *via* peptides for growth. The apparent biphasic growth, however, indicates that this mutant needs to make a critical phenotypic adaption for continued replication, which appears to be energy costly and ultimately cause cell death. Nonetheless, these data suggest that the polyamine spermidine serves an important role for the overall fitness and persistence of SCHU S4. In contrast, the deletion of *speE* from the LVS chromosome may reduce energy expenditure for the expression of this gene, since most of the genes involved in *spe*/*agu* metabolic pathways are truncated in this subpopulation. These findings indicate that type B strains have adapted to the absence of an intact *spe*/*agu* arginine catabolism pathway, whereas *de novo* biosynthesis of spermidine by *F. tularensis* A.I SCHU S4 provides a fitness advantage and promotes faster replication.

In summary, our findings show for the first time that arginine catabolism and polyamine biosynthesis differ considerably within the *F. tularensis* clades. The numerous IS elements within this species have contributed to genomic rearrangements and decay, as well as metabolic differences within the subpopulations. The broad host range and environmental conditions encountered by this facultative intracellular pathogen requires nutritional plasticity to persist. Although most intracellular pathogens have limited metabolic capacities, we propose that the retention of the polyamine biosynthesis pathway in the A.I subpopulation provides a fitness advantage that contributes to the persistence of this hypervirulent clade. Moreover, the loss of genes needed to produce agmatine, putrescine, and spermidine *de novo* in the *F. tularensis* A.II and B clades suggests that compensatory mechanisms exist to provide vital polyamines. Our current studies are focused on evaluating the contribution and regulation of arginine, methionine, and polyamine metabolism in *F. tularensis*, including during an intracellular infection, to better understand *F. tularensis* pathobiology.

## Data Availability Statement

The datasets presented in this study can be found in online repositories. The names of the repository/repositories and accession number(s) can be found at: NCBI BioProject—PRJNA817369, NCBI GEO—GSE202948.

## Author Contributions

YY and ML designed the experiments. YY, BG, and ML performed the experiments. BP and ML analyzed the transcriptome and proteome data. ML drafted the manuscript. All authors contributed to the article and approved the submitted version.

## Funding

This study was supported in part by a grant from the Nebraska Research Initiative (NRI) to ML and TH and NIH grant R35GM119770 to TH. The UNMC Genomics Core Facility receives partial support from the National Institute for General Medical Science (NIGMS) INBRE P20GM103427-19, as well as the National Cancer Institute and The Fred and Pamela Buffett Cancer Center Support grant P30CA036727. The contents of this publication are the sole responsibility of the authors and do not necessarily represent the views of any institution or agency.

## Conflict of Interest

The authors declare that the research was conducted in the absence of any commercial or financial relationships that could be construed as a potential conflict of interest.

## Publisher’s Note

All claims expressed in this article are solely those of the authors and do not necessarily represent those of their affiliated organizations, or those of the publisher, the editors and the reviewers. Any product that may be evaluated in this article, or claim that may be made by its manufacturer, is not guaranteed or endorsed by the publisher.
