# Supplementary material for: Arginine Catabolism and Polyamine Biosynthesis Pathway Disparities Within Francisella tularensis Subpopulations
Source: Front Microbiol. 2022 Jun 20;13:890856. doi: 10.3389/fmicb.2022.890856 (PMC9251427; doi:10.3389/fmicb.2022.890856)
Supplement: Supplementary file 1 [file Data_Sheet_1.PDF]

SUPPLEMENTARY MATERIAL

Table S1. Oligonucleotides used in this study.

Locus tag	Gene	Oligonucleotide sequence (5'-3')	Usage[a]
FTT_0430	*speH*	GGTAAATGGCCTGAGCATAAC	RT-qPCR (F1)
FTT_0430	*speH*	CCTCGACTACTTCACCTCTTC	RT-qPCR (R1)
FTT_0431	*speE*	GATGGTGGTATGCTTAGGGAAG	RT-qPCR (F1)
FTT_0431	*speE*	TACCGCTCTTAAGAAATCTCTGTG	RT-qPCR (R1)
FTT_0432	*speA*	GAATATTATAACTGTGATATGCCAAGC	RT-qPCR (F1)
FTT_0432	*speA*	GTGGTAAGTAACTCTGTAGTTCAC	RT-qPCR (R1)
FTT_0434	*aguA*	AATGATGAGAACTTTGCACGCC	RT-qPCR (F1)
FTT_0434	*aguA*	CCAAATGCTGGCATAATTATAGCG	RT-qPCR (R1)
FTT_0435	*aguB*	TGATTGATGCGGATGGTTCG	RT-qPCR (F1)
FTT_0435	*aguB*	TCATAAAGCTCAGGGCGACG	RT-qPCR (R1)
FTT_0149c	*metK*	TTGCATGATGCTGTGATTGAGG	RT-qPCR (F1)
FTT_0149c	*metK*	GCACCACCACCATGATGAG	RT-qPCR (R1)
FTT_0901	*lpnA*	CTTTATCAATCGCAGGTTTAGCG	RT-qPCR (F1)
FTT_0901	*lpnA*	TGAATCAGAAGCGATTACTTCTTTG	RT-qPCR (R1)
FTT_0430	*speH*	GTAACAATGTTGCTTTAGCTTTCTC	RT-qPCR (R2)
FTT_0431	*speE*	GAGTGATCTACTTTCAAATAGATGAG	RT-qPCR (F2)
FTT_0435	*aguB*	CAAATAGATATGCAACTGAGGCAAATG	RT-qPCR (F2)
FTT_0431	*speE*	AATGAGCTTGGTCTAAGTGCGCCCCAAATAG	*speE* deletion
FTT_0431	*speE*	TAATAATAACTCGGACGTAGCTATGTTATAGATACTCTAAAGAAGTAATAAC	*speE* deletion
FTT_0431	*speE*	GTAAATATCAAGCATGTCATAGCTGTTTCCTGTG	*speE* deletion
FTT_0431	*speE*	GCTACGTCCGAGTTATTATTATTTTTTATTATTAATATTAGCTATCATTAATTTACTAC	*speE* deletion
FTT_0431	*speE*	GCTATGACATGCTTGATATTTACATTGTTGTCTAGAAG	*speE* deletion
FTT_0431	*speE*	GCACTTAGACCAAGCTCATTACCCTGTTATCC	*speE* deletion
FTT_0431	*speE*	ATAGCTAGCGATTTCTAGATTTAAGAAGGAGATATACATATGCTCGAGATAGCTAATATTAATA ATAAAAAAATATTTCATG	*speE* expression
FTT_0431	*speE*	GGCGGATCCTTATTACTTCTTTAGAGTATCTATAAC	*speE* expression
FTT_0431	*speE*	ATAGCTAGCGATTTCTAGATTTAAGAAGGAGATATACATATGCTCGAGATAGCTAATATTAATA ATAAAAAAATATTTCATG	*speE* expression
FTT_0431	*speE*	GGCGGATCCTTATTACTTCTTTAGAGTATCTATAAC	*speE* expression
FTT_0430	*speH*	CAAACAGCTACAGTGTTAATATGTG	DNA sequencing
FTT_0429	*speA*	GGATATTAGCAACTACTTTATCAGTTG	DNA sequencing
FTT_0431	*speE*	AGATTGTTCTAAGATGCATACAAATTC	DNA sequencing

[a] F, Forward primer; R, Reverse primer.

SUPPLEMENTARY MATERIAL

Figure S1. Nucleotide sequence alignment of *speHEA* and *aguAB* operons in representative *Francisella tularensis* strains from the A.I, A.II, and B subpopulations. Representatives from each *F. tularensis* subpopulation includes subtype A.I strains SCHU S4 and MA00-2987, subtype A.II strains WY96-3418 and WY-00W4114, and type B strains FSC200 and LVS. Alignments were generated using Clustal Omega.

```
Ft_TypeB_FSC200              acaatttgtttttaagctgataacttatgcaatctgtttgtagcaaataacataaaacaa    60
Ft_TypeB_LVS                 acaatttgtttttaagctgataacttatgcaatctgtttgtagcaaataacataaaacaa    60
Ft_TypeAI_SCHU_S4            acaatttgtttttaagctgataacttatgcaagctgtttgtagcaaataacataaaacaa    60
Ft_TypeAI_MA00-2987          acaatttgtttttaagctgataacttatgcaagctgtttgtagcaaataacataaaacaa    60
Ft_TypeAII_WY96-3418         acaatttgtttttaagctgataacttatgcaagctgtttgtagcaaataacataaaacaa    60
Ft_TypeAII_WY-00W4114        acaatttgtttttaagctgataacttatgcaagctgtttgtagcaaataacataaaacaa    60
                             ******************************** ***************************

Ft_TypeB_FSC200              agaggatttatggctttaatttaatatagcacttaggctttttgactagaaaaaagtcta    120
Ft_TypeB_LVS                 agaggatttatggctttaatttaatatagcacttaggctttttgactagaaaaaagtcta    120
Ft_TypeAI_SCHU_S4            agaggatttatggctttaatttaatatagcacttaggctttttgactagaaaaaagtcta    120
Ft_TypeAI_MA00-2987          agaggatttatggctttaatttaatatagcacttaggctttttgactagaaaaaagtcta    120
Ft_TypeAII_WY96-3418         agaggatttatggctttaatttaatatagcacttaggctttttgactagaaaaaagtcta    120
Ft_TypeAII_WY-00W4114        agaggatttatggctttaatttaatatagcacttaggctttttgactagaaaaaagtcta    120
                             ************************************************************

Ft_TypeB_FSC200              agtgcgccccaaataggagatgaaaaatgatcattcaaaatactacagctatcctagata    180
Ft_TypeB_LVS                 agtgcgccccaaataggagatgaaaaatgatcattcaaaatactacagctatcctagata    180
Ft_TypeAI_SCHU_S4            agtgcgccccaaataggagatgaaaaatgatcattcaaaatactacagctatcctagata    180
Ft_TypeAI_MA00-2987          agtgcgccccaaataggagatgaaaaatgatcattcaaaatactacagctatcctagata    180
Ft_TypeAII_WY96-3418         agtgcgccccaaataggagatgaaaaatgatcattcaaaatactacagctatcctagata    180
Ft_TypeAII_WY-00W4114        agtgcgccccaaataggagatgaaaaatgatcattcaaaatactacagctatcctagata    180
                             ************************************************************

Ft_TypeB_FSC200              gcaacttaaaaaataaagaaatgggttcaggtaaatggcctgagcataacaatcaaatct    240
Ft_TypeB_LVS                 gcaacttaaaaaataaagaaatgggttcaggtaaatggcctgagcataacaatcaaatct    240
Ft_TypeAI_SCHU_S4            gcaacttaaaaaataaagaaatgggttcaggtaaatggcctgagcataacaatcaaatct    240
Ft_TypeAI_MA00-2987          gcaacttaaaaaataaagaaatgggttcaggtaaatggcctgagcataacaatcaaatct    240
Ft_TypeAII_WY96-3418         gcaacttaaaaaataaagaagtgggttcaggtaaatggcctgagcataacaatcaaatct    240
Ft_TypeAII_WY-00W4114        gcaacttaaaaaataaagaagtgggttcaggtaaatggcctgagcataacaatcaaatct    240
                             ******************** ***************************************
```

```
Ft_TypeB_FSC200              gtgataaccttcatcaagatcactttattcaagaaagtgtaaggtacgcgggaaatcatt    300
Ft_TypeB_LVS                 gtgataaccttcatcaagatcactttattcaagaaagtgtaaggtacgcgggaaatcatt    300
Ft_TypeAI_SCHU_S4            gtgataaccttcatcaagatcattttattcaagaaagtgtaaggtacgcgggaaatcatt    300
Ft_TypeAI_MA00-2987          gtgataaccttcatcaagatcattttattcaagaaagtgtaaggtacgcgggaaatcatt    300
Ft_TypeAII_WY96-3418         gtgataaccttcatcaagatcactttattcaagaaagtgtaaggtacgcgggaaatcatt    300
Ft_TypeAII_WY-00W4114        gtgataaccttcatcaagatcactttattcaagaaagtgtaaggtacgcgggaaatcatt    300
                             ********************** *************************************

Ft_TypeB_FSC200              taatagtcgacttttggggtgctagtaaattagattgccttgagactatggaagtagcaa    360
Ft_TypeB_LVS                 taatagtcgacttttggggtgctagtaaattagattgccttgagactatggaagtagcaa    360
Ft_TypeAI_SCHU_S4            taatagtcgacttttggggtgctagtaaattagattgccttgagactatggaagtagcaa    360
Ft_TypeAI_MA00-2987          taatagtcgacttttggggtgctagtaaattagattgccttgagactatggaagtagcaa    360
Ft_TypeAII_WY96-3418         taatagtcgacttttggggtgctagtaaattagattgccttgagactatggaagtagcaa    360
Ft_TypeAII_WY-00W4114        taatagtcgacttttggggtgctagtaaattagattgccttgagactatggaagtagcaa    360
                             ************************************************************

Ft_TypeB_FSC200              tgcgtgaagcagttgagaaagctaaagcaacattgttacacattcaccttcaccatttta    420
Ft_TypeB_LVS                 tgcgtgaagcagttgagaaagctaaagcaacattgttacacattcaccttcaccatttta    420
Ft_TypeAI_SCHU_S4            tgcgtgaagcagttgagaaagctaaagcaacattgttacacattcaccttcaccatttta    420
Ft_TypeAI_MA00-2987          tgcgtgaagcagttgagaaagctaaagcaacattgttacacattcaccttcaccatttta    420
Ft_TypeAII_WY96-3418         tgcgtgaagcagttgagaaagctaaagcaacattgttacacattcaccttcaccatttta    420
Ft_TypeAII_WY-00W4114        tgcgtgaagcagttgagaaagctaaagcaacattgttacacattcaccttcaccatttta    420
                             ************************************************************

Ft_TypeB_FSC200              cacctaatggcggtatttctggagttgctgtcttagcagaatcacatataagtgttcaca    480
Ft_TypeB_LVS                 cacctaatggcggtatttctggagttgctgtcttagcagaatcacatataagtgttcaca    480
Ft_TypeAI_SCHU_S4            cacctaatggcggtatttctggagttgctgtcttagcagaatcacatataagtgttcaca    480
Ft_TypeAI_MA00-2987          cacctaatggcggtatttctggagttgctgtcttagcagaatcacatataagtgttcaca    480
Ft_TypeAII_WY96-3418         cacctaatggcggtatttctggagttgctgtcttagcagaatcacatataagtgttcaca    480
Ft_TypeAII_WY-00W4114        cacctaatggcggtatttctggagttgctgtcttagcagaatcacatataagtgttcaca    480
                             ************************************************************

Ft_TypeB_FSC200              cttggcctgagtatggttttgcagcttttgatatatttatgtgtggagattctaaaccta    540
Ft_TypeB_LVS                 cttggcctgagtatggttttgcagcttttgatatatttatgtgtggagattctaaaccta    540
Ft_TypeAI_SCHU_S4            cttggcctgagtatggttttgcagcttttgatatatttatgtgtggagattctaaaccta    540
Ft_TypeAI_MA00-2987          cttggcctgagtatggttttgcagcttttgatatatttatgtgtggagattctaaaccta    540
Ft_TypeAII_WY96-3418         cttggcctgagtatggttttgcagcttttgatatatttatgtgtggagattctaaaccta    540
Ft_TypeAII_WY-00W4114        cttggcctgagtatggttttgcagcttttgatatatttatgtgtggagattctaaaccta    540
                             ************************************************************
```

```
Ft_TypeB_FSC200            ataaagcaatagaagttttagaaagttattttaagcctaaaacaaataatgtaagtaagt    600
Ft_TypeB_LVS               ataaagcaatagaagttttagaaagttattttaagcctaaaacaaataatgtaagtaagt    600
Ft_TypeAI_SCHU_S4          ataaagcaatagaagttttagaaagttattttaagcctaaaacaaataatgtaagtaagt    600
Ft_TypeAI_MA00-2987        ataaagcaatagaagttttagaaagttattttaagcctaaaacaaataatgtaagtaagt    600
Ft_TypeAII_WY96-3418       ataaagcaatagaagttttagaaagttattttaagcctaaaacaaataatgtaagtaagt    600
Ft_TypeAII_WY-00W4114      ataaagcaatagaagttttagaaagttattttaagcctaaaacaaataatgtaagtaagt    600
                           ************************************************************

Ft_TypeB_FSC200            ttagaagaggtgaagtagtcgaggtagtaaattaatgatagctaatattaataataaaaa    660
Ft_TypeB_LVS               ttagaagaggtgaagtagtcgaggtagtaaattaatgatagctaatattaataataaaaa    660
Ft_TypeAI_SCHU_S4          ttagaagaggtgaagtagtcgaggtagtaaattaatgatagctaatattaataataaaaa    660
Ft_TypeAI_MA00-2987        ttagaagaggtgaagtagtcgaggtagtaaattaatgatagctaatattaataataaaaa    660
Ft_TypeAII_WY96-3418       ttagaagaggtgaagtagtcgaggtagtaaattaatgatagctaatattaataataaaaa    660
Ft_TypeAII_WY-00W4114      ttagaagaggtgaagtagtcgaggtagtaaattaatgatagctaatattaataataaaaa    660
                           ************************************************************
Ft_TypeB_FSC200            aatatttcatgaaactttgtaccatagctatcatcaaagtatagcaatttctgaaatatt    720
Ft_TypeB_LVS               aatatttcatgaaactttgtaccatagctatcatcaaagtatagcaatttctgaaatatt    720
Ft_TypeAI_SCHU_S4          aatatttcatgaaactttgtaccatagctatcatcaaagtatagcaatttctgaaatatt    720
Ft_TypeAI_MA00-2987        aatatttcatgaaactttgtaccatagctatcatcaaagtatagcaatttctgaaatatt    720
Ft_TypeAII_WY96-3418       aatatttcatgaaactttgtaccatagctatcatcaaagtatagcaatttctgaaatatt    720
Ft_TypeAII_WY-00W4114      aatatttcatgaaactttgtaccatagctatcatcaaagtatagcaatttctgaaatatt    720
                           ************************************************************

Ft_TypeB_FSC200            gtatgaacataaaacagattaccaacatctagtaatttttaataatccaatttttggtaa    780
Ft_TypeB_LVS               gtatgaacataaaacagattaccaacatctagtaatttttaataatccaatttttggtaa    780
Ft_TypeAI_SCHU_S4          gtatgaacataaaacagattaccaacatctagtaatttttaataatccaatttttggtaa    780
Ft_TypeAI_MA00-2987        gtatgaacataaaacagattaccaacatctagtaatttttaataatccaatttttggtaa    780
Ft_TypeAII_WY96-3418       gtatgaacataaaacagattaccaacatctagtaatttttaataatccaatttttggtaa    780
Ft_TypeAII_WY-00W4114      gtatgaacataaaacagattaccaacatctagtaatttttaataatccaatttttggtaa    780
                           ************************************************************

Ft_TypeB_FSC200            tgtcatggtcttggatggtattgtacagactactgaaaaagatgagtttatttatcatga    840
Ft_TypeB_LVS               tgtcatggtcttggatggtattgtacagactactgaaaaagatgagtttatttatcatga    840
Ft_TypeAI_SCHU_S4          tgtcatggtcttggatggtattgtacagactactgaaaaagatgagtttatttatcatga    840
Ft_TypeAI_MA00-2987        tgtcatggtcttggatggtattgtacagactactgaaaaagatgagtttatttatcatga    840
Ft_TypeAII_WY96-3418       tgtcatggtcttggatggtattgtacagactactgaaaaagatgagtttatttatcatga    840
Ft_TypeAII_WY-00W4114      tgtcatggtcttggatggtattgtacagactactgaaaaagatgagtttatttatcatga    840
                           ************************************************************
```

```
Ft_TypeB_FSC200              aatgttagtgcatgtaccagttatcgcgcatgggaatgttaaaaatatattaattattgg     900
Ft_TypeB_LVS                 aatgttagtgcatgtaccagttatcgcgcatgggaatgttaaaaatatattaattattgg     900
Ft_TypeAI_SCHU_S4            aatgttagtgcatgtaccagttatcgcgcatgggaatgttaaaaacatattaattattgg     900
Ft_TypeAI_MA00-2987          aatgttagtgcatgtaccagttatcgcgcatgggaatgttaaaaacatattaattattgg     900
Ft_TypeAII_WY96-3418         aatgttagtgcatgtaccagttatcgcgcatgggaatgttaaaaacatattaattattgg     900
Ft_TypeAII_WY-00W4114        aatgttagtgcatgtaccagttatcgcgcatgggaatgttaaaaacatattaattattgg     900
                             ********************************************* **************

Ft_TypeB_FSC200              tggcggcgatggtggtatgcttagggaagctcttagtcataaagctgtcgaatttgtaac     960
Ft_TypeB_LVS                 tggcggcgatggtggtatgcttagggaagctcttagtcataaagctgtcgaatttgtaac     960
Ft_TypeAI_SCHU_S4            tggcggcgatggtggtatgcttagggaagctcttagtcataaagctgtcgaatctgtaac     960
Ft_TypeAI_MA00-2987          tggcggcgatggtggtatgcttagggaagctcttagtcataaagctgtcgaatctgtaac     960
Ft_TypeAII_WY96-3418         tggcggcgatggtggtatgcttagggaagctcttagtcataaagctgtcgaatctgtaac     960
Ft_TypeAII_WY-00W4114        tggcggcgatggtggtatgcttagggaagctcttagtcataaagctgtcgaatctgtaac     960
                             **************************************************** *******

Ft_TypeB_FSC200              actagttgaaattgaccaagctgttatagatatgtgccaagagtactttccagggcattc     1020
Ft_TypeB_LVS                 actagttgaaattgaccaagctgttatagatatgtgccaagagtactttccagggcattc     1020
Ft_TypeAI_SCHU_S4            actagttgaaattgaccaagctgttatagatatgtgccaagagtactttccagggcattc     1020
Ft_TypeAI_MA00-2987          actagttgaaattgaccaagctgttatagatatgtgccaagagtactttccagggcattc     1020
Ft_TypeAII_WY96-3418         actagttgaaattgaccaagctgttatagatatgtgccaagagtactttccagggcattc     1020
Ft_TypeAII_WY-00W4114        actagttgaaattgaccaagctgttatagatatgtgccaagagtactttccagggcattc     1020
                             ************************************************************
Ft_TypeB_FSC200              aaaaggtgcttttgatcatcctaaagcgaaaatagttattcaagatggttgtgaatttgt     1080
Ft_TypeB_LVS                 aaaaggtgcttttgatcatcctaaagcgaaaatagttattcaagatggttgtgaatttgt     1080
Ft_TypeAI_SCHU_S4            aaaaggtgcttttgatcatcctaaagcgaaaatagttattcaagatggttgtgaatttgt     1080
Ft_TypeAI_MA00-2987          aaaaggtgcttttgatcatcctaaagcgaaaatagttattcaagatggttgtgaatttgt     1080
Ft_TypeAII_WY96-3418         aaaaggtgcttttgatcatcctaaagcgaaaatagttattcaagatggttgtgaatttgt     1080
Ft_TypeAII_WY-00W4114        aaaaggtgcttttgatcatcctaaagcgaaaatagttattcaagatggttgtgaatttgt     1080
                             ************************************************************

Ft_TypeB_FSC200              caaaaatccgcctaggaaatatgatttgattatttgtgatagtactgatccgattggtcc     1140
Ft_TypeB_LVS                 caaaaatccgcctaggaaatatgatttgattatttgtgatagtactgatccgattggtcc     1140
Ft_TypeAI_SCHU_S4            caaaaatccgcctaggaaatatgatttgattatttgtgatagtactgatccgattggtcc     1140
Ft_TypeAI_MA00-2987          caaaaatccgcctaggaaatatgatttgattatttgtgatagtactgatccgattggtcc     1140
Ft_TypeAII_WY96-3418         caaaaatccgcctaggaaatatgatttgattatttgtgatagtactgatccgattggtcc     1140
Ft_TypeAII_WY-00W4114        caaaaatccgcctaggaaatatgatttgattatttgtgatagtactgatccgattggtcc     1140
                             ************************************************************
```

```
Ft_TypeB_FSC200               aggagaggttttatttacctcaaaattttataaagactgtaaagaagctctaaatccagg     1200
Ft_TypeB_LVS                  aggagaggttttatttacctcaaaattttataaagactgtaaagaagctctaaatccagg     1200
Ft_TypeAI_SCHU_S4             aggagaggttttatttacctcaaaattttataaagactgtaaagaagctataaatccagg     1200
Ft_TypeAI_MA00-2987           aggagaggttttatttacctcaaaattttataaagactgtaaagaagctataaatccagg     1200
Ft_TypeAII_WY96-3418          aggagaggttttatttacctcaaaattttataaagactgtaaagaagctctaaatccaga     1200
Ft_TypeAII_WY-00W4114         aggagaggttttatttacctcaaaattttataaagactgtaaagaagctctaaatccaga     1200
                              ************************************************* ********* 

Ft_TypeB_FSC200               tggtataatggtaacccaaaatggagtgatctactttcaaatagatgagttaaagaaaac     1260
Ft_TypeB_LVS                  tggtataatggtaacccaaaatggagtgatctactttcaaatagatgagttaaagaaaac     1260
Ft_TypeAI_SCHU_S4             tggtataatggtaacccaaaatggagtgatctactttcaaatagatgagttaaagaaaac     1260
Ft_TypeAI_MA00-2987           tggtataatggtaacccaaaatggagtgatctactttcaaatagatgagttaaagaaaac     1260
Ft_TypeAII_WY96-3418          tggtataatggtaacccaaaatggagtgatctactttcaaatagatgagttaaagaaaac     1260
Ft_TypeAII_WY-00W4114         tggtataatggtaacccaaaatggagtgatctactttcaaatagatgagttaaagaaaac     1260
                              ************************************************************

Ft_TypeB_FSC200               tcttgagcgctttgaaccattatataaagatgtttctttttatactgcagcagttccaac     1320
Ft_TypeB_LVS                  tcttgagcgctttgaaccattatataaagatgtttctttttatactgcagcagttccaac     1320
Ft_TypeAI_SCHU_S4             tcttgagcgctttgaaccattatataaagatgtttctttttatactgcagcagttccaac     1320
Ft_TypeAI_MA00-2987           tcttgagcgctttgaaccattatataaagatgtttctttttatactgcagcagttccaac     1320
Ft_TypeAII_WY96-3418          tcttgagcgctttgaaccattatataaagatgtttctttttatactgcagcagttccaac     1320
Ft_TypeAII_WY-00W4114         tcttgagcgctttgaaccattatataaagatgtttctttttatactgcagcagttccaac     1320
                              ************************************************************

Ft_TypeB_FSC200               atatgtcggtggaagtatggcatttggttggggcactgatgaattatcctatcgtaatca     1380
Ft_TypeB_LVS                  atatgtcggtggaagtatggcatttggttggggcactgatgaattatcctatcgtaatca     1380
Ft_TypeAI_SCHU_S4             atatgtcggtggaagtatggcatttggttggggcactgatgaattatcctatcgtaatca     1380
Ft_TypeAI_MA00-2987           atatgtcggtggaagtatggcatttggttggggcactgatgaattatcctatcgtaatca     1380
Ft_TypeAII_WY96-3418          atatgtcggtggaagtatggcatttggttggggcactgatgaattatcctatcgtaatca     1380
Ft_TypeAII_WY-00W4114         atatgtcggtggaagtatggcatttggttggggcactgatgaattatcctatcgtaatca     1380
                              ************************************************************

Ft_TypeB_FSC200               cgatattcaagtaattgcacagagatttcttaagagcggtattaaaacaaaatactacaa     1440
Ft_TypeB_LVS                  cgatattcaagtaattgcacagagatttcttaagagcggtattaaaacaaaatactacaa     1440
Ft_TypeAI_SCHU_S4             cgatattcaagtaattgcacagagatttcttaagagcggtattaaaacaaaatactacaa     1440
Ft_TypeAI_MA00-2987           cgatattcaagtaattgcacagagatttcttaagagcggtattaaaacaaaatactacaa     1440
Ft_TypeAII_WY96-3418          cgatattcaagtaattgcacagagatttcttaagagcggtattaaaacaagatactacaa     1440
Ft_TypeAII_WY-00W4114         cgatattcaagtaattgcacagagatttcttaagagcggtattaaaacaagatactacaa     1440
                              ************************************************** *********
```

```
Ft_TypeB_FSC200            tccagctatacatattgctgcttttgctttaccacaatatgttatagatactctaaagaa    1500
Ft_TypeB_LVS               tccagctatacatattgctgcttttgctttaccacaatatgttatagatactctaaagaa    1500
Ft_TypeAI_SCHU_S4          tccagctatacatattgctgcttttgctttaccacaatatgttatagatactctaaagaa    1500
Ft_TypeAI_MA00-2987        tccagctatacatattgctgcttttgctttaccacaatatgttatagatactctaaagaa    1500
Ft_TypeAII_WY96-3418       tccagctatacatattgctgcttttgctttaccacaatatgttatagatactctaaagaa    1500
Ft_TypeAII_WY-00W4114      tccagctatacatattgctgcttttgctttaccacaatatgttatagatactctaaagaa    1500
                           ************************************************************

Ft_TypeB_FSC200            gtaataacttaacctcataagaaggtaagatgaacaaaaatttatttctaacagctaatc    1560
Ft_TypeB_LVS               gtaataacttaacctcataagaaggtaagatgaacaaaaatttatttctaacagctaatc    1560
Ft_TypeAI_SCHU_S4          gtaataacttaacctcataaggaggtaagatgaacaaaaatttatttctaacagctaatc    1560
Ft_TypeAI_MA00-2987        gtaataacttaacctcataaggaggtaagatgaacaaaaatttatttctaacagctaatc    1560
Ft_TypeAII_WY96-3418       gtaataacttaacctcataaggaggtaagatgaacaaaaatttatttctaacagctaatc    1560
Ft_TypeAII_WY-00W4114      gtaataacttaacctcataaggaggtaagatgaacaaaaatttatttctaacagctaatc    1560
                           ********************* **************************************

Ft_TypeB_FSC200            tatcagagtattttagctataatagtttaggtcaattatgtatcaactatgctgatggtt    1620
Ft_TypeB_LVS               tatcagagtattttagctataatagtttaggtcaattatgtatcaactatgctgatggtt    1620
Ft_TypeAI_SCHU_S4          tatcagagtattttagctataatagtttaggtcaattatgtatcaactatgctgatggtt    1620
Ft_TypeAI_MA00-2987        tatcagagtattttagctataatagtttaggtcaattatgtatcaactatgctgatggtt    1620
Ft_TypeAII_WY96-3418       tatcagagtattttagctataatagtttaggtcaattatgtatcaactatgctgatggtt    1620
Ft_TypeAII_WY-00W4114      tatcagagtattttagctataatagtttaggtcaattatgtatcaactatgctgatggtt    1620
                           ************************************************************

Ft_TypeB_FSC200            atagaaaaactattcctatcgtcgattttttgcaacaggcaaactttcagggagctacat    1680
Ft_TypeB_LVS               atagaaaaactattcctatcgtcgattttttgcaacaggcaaactttcagggagctacat    1680
Ft_TypeAI_SCHU_S4          atagaaaaactattcctatcgtcgattttttgcaacaggcaaactttcagggagctacat    1680
Ft_TypeAI_MA00-2987        atagaaaaactattcctatcgtcgattttttgcaacaggcaaactttcagggagctacat    1680
Ft_TypeAII_WY96-3418       atagaaaaactattcctatcgtcgattttttgcaacaggcaaactttcagggagctacat    1680
Ft_TypeAII_WY-00W4114      atagaaaaactattcctatcgtcgattttttgcaacaggcaaactttcagggagctacat    1680
                           ************************************************************

Ft_TypeB_FSC200            atccattggttttatattttgagcatatcttaaaaaactaacttgataaactttatacta    1740
Ft_TypeB_LVS               atccattggttttatattttgagcatatcttaaaaaactaacttgataaactttatacta    1740
Ft_TypeAI_SCHU_S4          atccattggttttatattttgagcatatcttaaaaaaccaacttgataaactttatacta    1740
Ft_TypeAI_MA00-2987        atccattggttttatattttgagcatatcttaaaaaaccaacttgataaactttatacta    1740
Ft_TypeAII_WY96-3418       atccattggttttatattttgagcatatcttaaaaaaccaacttgataaactttatacta    1740
Ft_TypeAII_WY-00W4114      atccattggttttatattttgagcatatcttaaaaaaccaacttgataaactttatacta    1740
                           ************************************** *********************
```

```
Ft_TypeB_FSC200                 gttttggaaaagctatagatgattttagctatacaggtaaatatagtttagcttatccga    1800
Ft_TypeB_LVS                    gttttggaaaagctatagatgattttagctatacaggtaaatatagtttagcttatccga    1800
Ft_TypeAI_SCHU_S4               gttttggaaaagctatagatgattttagctatacatgtaaatatagtttagcttatccga    1800
Ft_TypeAI_MA00-2987             gttttggaaaagctatagatgattttagctatacatgtaaatatagtttagcttatccga    1800
Ft_TypeAII_WY96-3418            gttttggaaaagctatagatgattttagctatacatgtaaatatagtttagcttatccga    1800
Ft_TypeAII_WY-00W4114           gttttggaaaagctatagatgattttagctatacatgtaaatatagtttagcttatccga    1800
                                *********************************** ************************

Ft_TypeB_FSC200                 taaaaataaatcctgaacaaaagattataaaggcaatttatgattatcatcaacataatc    1860
Ft_TypeB_LVS                    taaaaataaatcctgaacaaaagattataaaggcaatttatgattatcatcaacataatc    1860
Ft_TypeAI_SCHU_S4               taaaagtaaatcctgaacaaaagattataaaggcaatttatgattatcatcaacataatc    1860
Ft_TypeAI_MA00-2987             taaaagtaaatcctgaacaaaagattataaaggcaatttatgattatcatcaacataatc    1860
Ft_TypeAII_WY96-3418            taaaagtaaatcctgaacaaaagattataaaggcaatttatgattatcatcaacataatc    1860
Ft_TypeAII_WY-00W4114           taaaagtaaatcctgaacaaaagattataaaggcaatttatgattatcatcaacataatc    1860
                                ***** ******************************************************

Ft_TypeB_FSC200                 aagttagtttggattatgaggtaggtactaaatctgagtttttagctgtgctaagtacag    1920
Ft_TypeB_LVS                    aagttagtttggattatgaggtaggtactaaatctgagtttttagctgtgctaagtacag    1920
Ft_TypeAI_SCHU_S4               aagttagtttggattatgaggtaggtactaaatctgagtttttagctgtgctaagtacag    1920
Ft_TypeAI_MA00-2987             aagttagtttggattatgaggtaggtactaaatctgagtttttagctgtgctaagtacag    1920
Ft_TypeAII_WY96-3418            aagttagtttggattatgaggtaggtactaaatctgagtttttagctgtgctaagtacag    1920
Ft_TypeAII_WY-00W4114           aagttagtttggattatgaggtaggtactaaatctgagtttttagctgtgctaagtacag    1920
                                ************************************************************

Ft_TypeB_FSC200                 cagctaaaacacaaaagattatttgtaatggttttaaagatcttagtttttataaaatag    1980
Ft_TypeB_LVS                    cagctaaaacacaaaagattatttgtaatggttttaaagatcttagtttttataaaatag    1980
Ft_TypeAI_SCHU_S4               cagctaaaacacaaaagattatttgtaatggttttaaagatcttagtttttataaaatag    1980
Ft_TypeAI_MA00-2987             cagctaaaacacaaaagattatttgtaatggttttaaagatcttagtttttataaaatag    1980
Ft_TypeAII_WY96-3418            cagctaaaacacaaaagattatttgtaatggttttaaagatcttagtttttataaaatag    1980
Ft_TypeAII_WY-00W4114           cagctaaaacacaaaagattatttgtaatggttttaaagatcttagtttttataaaatag    1980
                                ************************************************************

Ft_TypeB_FSC200                 cacagctagctgcaaaaataggctataaaattataatagttattgaaaacattagtgaaa    2040
Ft_TypeB_LVS                    cacagctagctgcaaaaataggctataaaattataatagttattgaaaacattagtgaaa    2040
Ft_TypeAI_SCHU_S4               cacagctagctgcaaaaataggctataaaattataatagttattgaaaacattagtgaaa    2040
Ft_TypeAI_MA00-2987             cacagctagctgcaaaaataggctataaaattataatagttattgaaaacattagtgaaa    2040
Ft_TypeAII_WY96-3418            cacagctatctgcaaaaataggctataaaattataatagttattgaaaacattagtgaaa    2040
Ft_TypeAII_WY-00W4114           cacagctatctgcaaaaataggctataaaattataatagttattgaaaacattagtgaaa    2040
                                ******** ***************************************************
```

```
Ft_TypeB_FSC200            taaatattattcaggatcttctagacaacaatgtaaatatcaagtttgattttggcatta    2100
Ft_TypeB_LVS               taaatattattcaggatcttctagacaacaatgtaaatatcaagtttgattttggcatta    2100
Ft_TypeAI_SCHU_S4          taaatattattcaggatcttctagacaacaatgtaaatatcaagtttgattttggcatca    2100
Ft_TypeAI_MA00-2987        taaatattattcaggatcttctagacaacaatgtaaatatcaagtttgattttggcatca    2100
Ft_TypeAII_WY96-3418       taaatattattcaggatcttctagacaacaatgtaaatatcaagtttgattttggcatca    2100
Ft_TypeAII_WY-00W4114      taaatattattcaggatcttctagacaacaatgtaaatatcaagtttgattttggcatca    2100
                           ********************************************************** *

Ft_TypeB_FSC200            ggacaaagccttttgataattgcatacatacccaaaaatttggcttaagtttatcccaaa    2160
Ft_TypeB_LVS               ggacaaagccttttgataattgcatacatacccaaaaatttggcttaagtttatcccaaa    2160
Ft_TypeAI_SCHU_S4          ggacaaagccttttgataattgcatacatacccaaaaatttggcttaagtttatcccaaa    2160
Ft_TypeAI_MA00-2987        ggacaaagccttttgataattgcatacatacccaaaaatttggcttaagtttatcccaaa    2160
Ft_TypeAII_WY96-3418       ggacaaagccttttgataattgcatacatacccaaaaatttggcttaagtttatcccaaa    2160
Ft_TypeAII_WY-00W4114      ggacaaagccttttgataattgcatacatacccaaaaatttggcttaagtttatcccaaa    2160
                           ************************************************************

Ft_TypeB_FSC200            tcaacgagtgtattaatttcttgaaaactaatcagctccaacaaaaactacttttattgc    2220
Ft_TypeB_LVS               tcaacgagtgtattaatttcttgaaaactaatcagctccaacaaaaactacttttattgc    2220
Ft_TypeAI_SCHU_S4          tcaacgagtgtattaatttcttgaaaactaatcagctccaacaaaaactacttttattgc    2220
Ft_TypeAI_MA00-2987        tcaacgagtgtattaatttcttgaaaactaatcagctccaacaaaaactacttttattgc    2220
Ft_TypeAII_WY96-3418       tcaacgagtgtattaatttcttgaaaactaatcagctccaataaaaactacttttattgc    2220
Ft_TypeAII_WY-00W4114      tcaacgagtgtattaatttcttgaaaactaatcagctccaataaaaactacttttattgc    2220
                           ***************************************** ******************

Ft_TypeB_FSC200            acagtcatataggctctcagctaaagtcaactgataaagtagttgctaatatccaaagat    2280
Ft_TypeB_LVS               acagtcatataggctctcagctaaagtcaactgataaagtagttgctaatatccaaagat    2280
Ft_TypeAI_SCHU_S4          acagtcatataggctctcagataaagtcaactgataaagtagttgctaatatccaaagat    2280
Ft_TypeAI_MA00-2987        acagtcatataggctctcagataaagtcaactgataaagtagttgctaatatccaaagat    2280
Ft_TypeAII_WY96-3418       acagtcatataggctctcagataaaatcaactgataaagtagttgctaatatccaaagat    2280
Ft_TypeAII_WY-00W4114      acagtcatataggctctcagataaaatcaactgataaagtagttgctaatatccaaagat    2280
                           ******************** **** **********************************

Ft_TypeB_FSC200            taatgaggatttattcagaattaaaaagtgattttgtaaatcttgagtatattgattttg    2340
Ft_TypeB_LVS               taatgaggatttattcagaattaaaaagtgattttgtaaatcttgagtatattgattttg    2340
Ft_TypeAI_SCHU_S4          taatgaggatttattcagaattaaaaagtgattttgtaaatcttgagtatattgattttg    2340
Ft_TypeAI_MA00-2987        taatgaggatttattcagaattaaaaagtgattttgtaaatcttgagtatattgattttg    2340
Ft_TypeAII_WY96-3418       taatgaggatttattcagaattaaaaagtgattttgtaaatcttgagtatattgattttg    2340
Ft_TypeAII_WY-00W4114      taatgaggatttattcagaattaaaaagtgattttgtaaatcttgagtatattgattttg    2340
                           ************************************************************
```

```
Ft_TypeB_FSC200                   gtggtggtctagtagttaattatgatgaatatgatagaccaagttatgattttgatagtt     2400
Ft_TypeB_LVS                      gtggtggtctagtagttaattatgatgaatatgatagaccaagttatgattttgatagtt     2400
Ft_TypeAI_SCHU_S4                 gtggtggtctagcagttaattatgatgaatatgatagaccaagttatgattttgatagtt     2400
Ft_TypeAI_MA00-2987               gtggtggtctagcagttaattatgatgaatatgatagaccaagttatgattttgatagtt     2400
Ft_TypeAII_WY96-3418              gtggtggtctagcagttaattatgatgaatatgatagaccaagttatgattttgatagtt     2400
Ft_TypeAII_WY-00W4114             gtggtggtctagcagttaattatgatgaatatgatagaccaagttatgattttgatagtt     2400
                                  ************ ***********************************************

Ft_TypeB_FSC200                   atgctaaaaatattatcaaaaattgtaaatactatgctgaatattataactgtgatatgc     2460
Ft_TypeB_LVS                      atgctaaaaatattatcaaaaattgtaaatactatgctgaatattataactgtgatatgc     2460
Ft_TypeAI_SCHU_S4                 atgctaaaaatattatcaaaaattgtaaatactatgctgaatattataactgtgatatgc     2460
Ft_TypeAI_MA00-2987               atgctaaaaatattatcaaaaattgtaaatactatgctgaatattataactgtgatatgc     2460
Ft_TypeAII_WY96-3418              atgctaaaaatattatcaaaaattgtaaatactatgctgaatattataactgtgatatgc     2460
Ft_TypeAII_WY-00W4114             atgctaaaaatattatcaaaaattgtaaatactatgctgaatattataactgtgatatgc     2460
                                  ************************************************************

Ft_TypeB_FSC200                   caagcatagttacagagtctggaagagcaatcacagcagagtcaagtttgttaataacaa     2520
Ft_TypeB_LVS                      caagcatagttacagagtctggaagagcaatcacagcagagtcaagtttgttaataacaa     2520
Ft_TypeAI_SCHU_S4                 caagcatagttacagagtctggaagagcaatcacagcagagtcaagtttgttaataacaa     2520
Ft_TypeAI_MA00-2987               caagcatagttacagagtctggaagagcaatcacagcagagtcaagtttgttaataacaa     2520
Ft_TypeAII_WY96-3418              caagcatagttacagagtctggaagagcaatcacagcagagtcaagtttgttaataacaa     2520
Ft_TypeAII_WY-00W4114             caagcatagttacagagtctggaagagcaatcacagcagagtcaagtttgttaataacaa     2520
                                  ************************************************************

Ft_TypeB_FSC200                   aaccactgataaaacagcatgagtcagatattgagaatgaaatcctacatcagcagtggt     2580
Ft_TypeB_LVS                      aaccactgataaaacagcatgagtcagatattgagaatgaaatcctacatcagcagtggt     2580
Ft_TypeAI_SCHU_S4                 aaccactgataaaacagcatgagtcagatattgagaatgaaatcctacatcagcagtggt     2580
Ft_TypeAI_MA00-2987               aaccactgataaaacagcatgagtcagatattgagaatgaaatcctacatcagcagtggt     2580
Ft_TypeAII_WY96-3418              aaccactgataaaacagcatgagtcagatattgagaatgaaatcctacatcagcagtggt     2580
Ft_TypeAII_WY-00W4114             aaccactgataaaacagcatgagtcagatattgagaatgaaatcctacatcagcagtggt     2580
                                  ************************************************************

Ft_TypeB_FSC200                   tagatagagaagtgtcgcttagtgaactacagagttacttaccacaaaatcaaaaaaata     2640
Ft_TypeB_LVS                      tagatagagaagtgtcgcttagtgaactacagagttacttaccacaaaatcaaaaaaata     2640
Ft_TypeAI_SCHU_S4                 tagatagagaagtgtcgcttggtgaactacagagttacttaccacaaaatcaaaaaaata     2640
Ft_TypeAI_MA00-2987               tagatagagaagtgtcgcttggtgaactacagagttacttaccacaaaatcaaaaaaata     2640
Ft_TypeAII_WY96-3418              tagatagagaagtgtcgcttggtgaactacagagttacttaccacaaaatcaa-aaaata     2639
Ft_TypeAII_WY-00W4114             tagatagagaagtgtcgcttggtgaactacagagttacttaccacaaaatcaa-aaaata     2639
                                  ******************** ******************************** ******
```

```
Ft_TypeB_FSC200            ctcagcaagcgtggttaaatttttcaatatttcaatcattacttgatcattggggcatag    2700
Ft_TypeB_LVS               ctcagcaagcgtggttaaatttttcaatatttcaatcattacttgatcattggggcatag    2700
Ft_TypeAI_SCHU_S4          ctcagcaagcgtggttaaatttttcaatatttcaatcattacctgatcattggggcatag    2700
Ft_TypeAI_MA00-2987        ctcagcaagcgtggttaaatttttcaatatttcaatcattacctgatcattggggcatag    2700
Ft_TypeAII_WY96-3418       ctcagcaagcgtggttaaatttttcaatatttcaatcattacctgatcattggggcatag    2699
Ft_TypeAII_WY-00W4114      ctcagcaagcgtggttaaatttttcaatatttcaatcattacctgatcattggggcatag    2699
                           ***************************************** ******************

Ft_TypeB_FSC200            accaaaaatttccgatacttcctttggagtttttaatagctatgtgacctcagaagtta    2760
Ft_TypeB_LVS               accaaaaatttccgatacttcctttggagtttttaatagctatgtgacctcagaagtta    2760
Ft_TypeAI_SCHU_S4          accaaaaatttccgatacttcctttggagtttttaatagctatgtgacctcagaagtta    2760
Ft_TypeAI_MA00-2987        accaaaaatttccgatacttcctttggagtttttaatagctatgtgacctcagaagtta    2760
Ft_TypeAII_WY96-3418       accaaaaatttccgatacttcctttggagtttttaatagctatgtgacctcagaagtta    2759
Ft_TypeAII_WY-00W4114      accaaaaatttccgatacttcctttggagtttttaatagctatgtgacctcagaagtta    2759
                           ************************************************************

Ft_TypeB_FSC200            aactttatgatatttcatatgatgttgatggtgtagtaaagtcaacttcagaatatattg    2820
Ft_TypeB_LVS               aactttatgatatttcatatgatgttgatggtgtagtaaagtcaacttcagaatatattg    2820
Ft_TypeAI_SCHU_S4          aactttatgatatttcatgtgacgttgatggtgtagtaaagtcaacttcagaatatattg    2820
Ft_TypeAI_MA00-2987        aactttatgatatttcatgtgacgttgatggtgtagtaaagtcaacttcagaatatattg    2820
Ft_TypeAII_WY96-3418       aactttatgatatttcatgtgacgttgatggtgtagtaaagtcaacttcagaatatattg    2819
Ft_TypeAII_WY-00W4114      aactttatgatatttcatgtgacgttgatggtgtagtaaagtcaacttcagaatatattg    2819
                           ****************** *** *************************************

Ft_TypeB_FSC200            agatagctactgataatattgaatatattgtttttatgtgtgtgggtgcttatcagggga    2880
Ft_TypeB_LVS               agatagctactgataatattgaatatattgtttttatgtgtgtgggtgcttatcagggga    2880
Ft_TypeAI_SCHU_S4          agatagctactgataatattgaatatattgtttttatgtgtgtgggtgcttatcagggga    2880
Ft_TypeAI_MA00-2987        agatagctactgataatattgaatatattgtttttatgtgtgtgggtgcttatcagggga    2880
Ft_TypeAII_WY96-3418       agatagctactgataatattgaatatattgtttttatgtgtgtgggtgcttatcagggga    2879
Ft_TypeAII_WY-00W4114      agatagctactgataatattgaatatattgtttttatgtgtgtgggtgcttatcagggga    2879
                           ************************************************************

Ft_TypeB_FSC200            tgttaagcgctaagcataatatgcttggaaatattagtgcggtgaatatatatatagatg    2940
Ft_TypeB_LVS               tgttaagcgctaagcataatatgcttggaaatattagtgcggtgaatatatatatagatg    2940
Ft_TypeAI_SCHU_S4          tgttaagcgctaagcataatatgcttggaaatattagtgcggtgaatatat--atagatg    2938
Ft_TypeAI_MA00-2987        tgttaagcgctaagcataatatgcttggaaatattagtgcggtgaatatat--atagatg    2938
Ft_TypeAII_WY96-3418       tgttaagcgctaagcataatatgcttggaaatattagtgcggtgaatatatatatagatg    2939
Ft_TypeAII_WY-00W4114      tgttaagcgctaagcataatatgcttggaaatattagtgcggtgaatatatatatagatg    2939
                           ***************************************************  *******
```

```
Ft_TypeB_FSC200            aaaatagaaaagtaaaagtatctgtaaaagctgcggaaaattactattcattgttagatc   3000
Ft_TypeB_LVS               aaaatagaaaagtaaaagtatctgtaaaagctgcggaaaattactattcattgttagatc   3000
Ft_TypeAI_SCHU_S4          aaaatagaaaagtaaaagtatctgtaaaagctgcggaaaattactattcattgttagatc   2998
Ft_TypeAI_MA00-2987        aaaatagaaaagtaaaagtatctgtaaaagctgcggaaaattactattcattgttagatc   2998
Ft_TypeAII_WY96-3418       aaaatagaaaagtaaaagtatctgtaaaagctgcggaaaattactattcattgttagatc   2999
Ft_TypeAII_WY-00W4114      aaaatagaaaagtaaaagtatctgtaaaagctgcggaaaattactattcattgttagatc   2999
                           ************************************************************

Ft_TypeB_FSC200            aatatggttataactctctgaggatacagtcaaatttaaaaagatactattattatcacc   3060
Ft_TypeB_LVS               aatatggttataactctctgaggatacagtcaaatttaaaaagatactattattatcacc   3060
Ft_TypeAI_SCHU_S4          aatatggttataactctctgaggatacagtcaaatttaaaaagatactattattatcacc   3058
Ft_TypeAI_MA00-2987        aatatggttataactctctgaggatacagtcaaatttaaaaagatactattattatcacc   3058
Ft_TypeAII_WY96-3418       aatatagttataactctctgaggatacagtcaaatttaaaaagatactattattatcacc   3059
Ft_TypeAII_WY-00W4114      aatatagttataactctctgaggatacagtcaaatttaaaaagatactattattatcacc   3059
                           ***** ******************************************************

Ft_TypeB_FSC200            aagaggaaataaaccaagaggaagaagaatttttagacaatttatttattgataatccat   3120
Ft_TypeB_LVS               aagaggaaataaaccaagaggaagaagaatttttagacaatttatttattgataatccat   3120
Ft_TypeAI_SCHU_S4          aagaggagataaaccaagaggaagaagaatttttagacaatttatttattgataatccat   3118
Ft_TypeAI_MA00-2987        aagaggagataaaccaagaggaagaagaatttttagacaatttatttattgataatccat   3118
Ft_TypeAII_WY96-3418       aagaggaaataaaccaagaggaagaagaatttttagacaatttatttattgataagccat   3119
Ft_TypeAII_WY-00W4114      aagaggaaataaaccaagaggaagaagaatttttagacaatttatttattgataagccat   3119
                           ******* *********************************************** ****

Ft_TypeB_FSC200            atatgggcgagataactagtcaacaaggaggaataaattatgctattatggccggctgaa   3180
Ft_TypeB_LVS               atatgggcgagataactagtcaacaaggaggaataaattatgctattatggccggctgaa   3180
Ft_TypeAI_SCHU_S4          atatgggcgagataactagtcaacaaggagggataaattatgctattatggccggctgaa   3178
Ft_TypeAI_MA00-2987        atatgggcgagataactagtcaacaaggagggataaattatgctattatggccggctgaa   3178
Ft_TypeAII_WY96-3418       atatgggcgagataactagtcaacaaggagggataaattatgctattatggccggctgaa   3179
Ft_TypeAII_WY-00W4114      atatgggcgagataactagtcaacaaggagggataaattatgctattatggccggctgaa   3179
                           ******************************* ****************************

Ft_TypeB_FSC200            tgggaggagcatagtgcaacttggatgatctggccagcacgtatagatatgtggccaaat   3240
Ft_TypeB_LVS               tgggaggagcatagtgcaacttggatgatctggccagcacgtatagatatgtggccaaat   3240
Ft_TypeAI_SCHU_S4          tgggaggagcatagtgcaacttggatgatctggccagcacgtatagatatgtggccaaat   3238
Ft_TypeAI_MA00-2987        tgggaggagcatagtgcaacttggatgatctggccagcacgtatagatatgtggccaaat   3238
Ft_TypeAII_WY96-3418       tgggaggagcatagtgcaacttggatgatctggccagcacgtatagatatgtggccaaat   3239
Ft_TypeAII_WY-00W4114      tgggaggagcatagtgcaacttggatgatctggccagcacgtatagatatgtggccaaat   3239
                           ************************************************************
```

```
Ft_TypeB_FSC200                    ataacaaaagcctatgagatatatgctaaagttgctaacactatcgctaaatatgagcct      3300
Ft_TypeB_LVS                       ataacaaaagcctatgagatatatgctaaagttgctaacactatcgctaaatatgagcct      3300
Ft_TypeAI_SCHU_S4                  ataacaaaagcctatgagatatatgctaaagttgctaacactatcgctaaatatgagcct      3298
Ft_TypeAI_MA00-2987                ataacaaaagcctatgagatatatgctaaagttgctaacactatcgctaaatatgagcct      3298
Ft_TypeAII_WY96-3418               ataacaaaagcctatgagatatatgctaaagttgctaacactatcgctaaatatgagcct      3299
Ft_TypeAII_WY-00W4114              ataacaaaagcctatgagatatatgctaaagttgctaacactatcgctaaatatgagcct      3299
                                   ************************************************************

Ft_TypeB_FSC200                    gtaaatatggtagtaaatcagcatcagttagatattgcaaaaaattatcttgtaaaaaac      3360
Ft_TypeB_LVS                       gtaaatatggtagtaaatcagcatcagttagatattgcaaaaaattatcttgtaaaaaac      3360
Ft_TypeAI_SCHU_S4                  gtaaatatggtagtaaatcagcatcagttagatattgcaaaaaaatatcttggaaaaaac      3358
Ft_TypeAI_MA00-2987                gtaaatatggtagtaaatcagcatcagttagatattgcaaaaaaatatcttggaaaaaac      3358
Ft_TypeAII_WY96-3418               gtaaatatggtagtaaatcagcatcagttagatattgcaaaaaattatcttggaaaaaac      3359
Ft_TypeAII_WY-00W4114              gtaaatatggtagtaaatcagcatcagttagatattgcaaaaaattatcttggaaaaaac      3359
                                   ******************************************** ******* *******

Ft_TypeB_FSC200                    ataaccctgataagtgaagtagtagatgatagttgggctagagatattatgccaattttt      3420
Ft_TypeB_LVS                       ataaccctgataagtgaagtagtagatgatagttgggctagagatattatgccaattttt      3420
Ft_TypeAI_SCHU_S4                  atgaccctgataagtgaagcagtagatgatagttgggctagagatattatgccaattttt      3418
Ft_TypeAI_MA00-2987                atgaccctgataagtgaagcagtagatgatagttgggctagagatattatgccaattttt      3418
Ft_TypeAII_WY96-3418               atgaccctgataagtgaagcagtagatgatagttgggctagagatattatgccaattttt      3419
Ft_TypeAII_WY-00W4114              atgaccctgataagtgaagcagtagatgatagttgggctagagatattatgccaattttt      3419
                                   ** **************** ****************************************

Ft_TypeB_FSC200                    tcatttaaagcagataaactcattgcaaataattttgattttaactgttggagtaataag      3480
Ft_TypeB_LVS                       tcatttaaagcagataaactcattgcaaataattttgattttaactgttggagtaataag      3480
Ft_TypeAI_SCHU_S4                  tcatttaaagcagataaactcattgcaaataattttgattttaactgctggagtaataag      3478
Ft_TypeAI_MA00-2987                tcatttaaagcagataaactcattgcaaataattttgattttaactgctggggtaataag      3478
Ft_TypeAII_WY96-3418               tcatttaaagcagataaactcattgcaaataattttgattttaactgctggggtaataag      3479
Ft_TypeAII_WY-00W4114              tcatttaaagcagataaactcattgcaaataattttgattttaactgctggggtaataag      3479
                                   *********************************************** *** ********

Ft_TypeB_FSC200                    ttttcaccttttgataacgatagaagacttaaaaatgatattgcaaaacaacaaaaatgg      3540
Ft_TypeB_LVS                       ttttcaccttttgataacgatagaagacttaaaaatgatattgcaaaacaacaaaaatgg      3540
Ft_TypeAI_SCHU_S4                  ttttcaccttttgataacgatagaagacttaaaaatgatattgcaaaacaacaaaaatgg      3538
Ft_TypeAI_MA00-2987                ttttcaccttttgataacgatagaagacttaaaaatgatattgcaaaacaacaaaaatgg      3538
Ft_TypeAII_WY96-3418               ttttcaccttttgataacgatagaagacttaaaaatgatattgcaaaacaacaaaaatgg      3539
Ft_TypeAII_WY-00W4114              ttttcaccttttgataacgatagaagacttaaaaatgatattgcaaaacaacaaaaatgg      3539
                                   ************************************************************
```

```
Ft_TypeB_FSC200              caggttaattcttctaaaatgattttagagggtggagcagtacattcaaatggtcaagga    3600
Ft_TypeB_LVS                 caggttaattcttctaaaatgattttagagggtggagcagtacattcaaatggtcaagga    3600
Ft_TypeAI_SCHU_S4            caggttaattcttctaaaatgattttagagggtggagcagtacattcaaatggtcaagga    3598
Ft_TypeAI_MA00-2987          caggttaattcttctaaaatgattttagagggtggagcagtacattcaaatggtcaagga    3598
Ft_TypeAII_WY96-3418         caggttaattcttctaaaatgattttagagggtggagcagtacattcaaatggtcaagga    3599
Ft_TypeAII_WY-00W4114        caggttaattcttctaaaatgattttagagggtggagcagtacattcaaatggtcaagga    3599
                             ************************************************************

Ft_TypeB_FSC200              gttttattaacaaccaaggaatgcttactaaatttaaaccgtaatccaaatatgcaaaaa    3660
Ft_TypeB_LVS                 gttttattaacaaccaaggaatgcttactaaatttaaaccgtaatccaaatatgcaaaaa    3660
Ft_TypeAI_SCHU_S4            gttttattaacaaccaaggaatgcttactaaatttaaaccgtaatccaaatatgcaaaaa    3658
Ft_TypeAI_MA00-2987          gttttattaacaaccaaggaatgcttactaaatttaaaccgtaatccaaatatgcaaaaa    3658
Ft_TypeAII_WY96-3418         gttttattaacaaccaaggaatgcttactaaatttaaaccgtaatccaaatatgcaaaaa    3659
Ft_TypeAII_WY-00W4114        gttttattaacaaccaaggaatgcttactaaatttaaaccgtaatccaaatatgcaaaaa    3659
                             ************************************************************

Ft_TypeB_FSC200              gaacaaattgaaagtgagttaatcagtattttgggagttaaaaaaattctttggctacca    3720
Ft_TypeB_LVS                 gaacaaattgaaagtgagttaatcagtattttgggagttaaaaaaattctttggctacca    3720
Ft_TypeAI_SCHU_S4            gagcaaattgaaagtgagttaatcagtattttgggagttaaaaaaattctttggctacca    3718
Ft_TypeAI_MA00-2987          gagcaaattgaaagtgagttaatcagtattttgggagttaaaaaaattctttggctacca    3718
Ft_TypeAII_WY96-3418         gagcaaattgaaagtgagttaatcagtattttgggagttaaaaaaattctttggctacca    3719
Ft_TypeAII_WY-00W4114        gagcaaattgaaagtgagttaatcagtattttgggagttaaaaaaattctttggctacca    3719
                             ** *********************************************************

Ft_TypeB_FSC200              tatggtgtagcaggtgattttgatacagatggtcatgttgataacgttgcttgctttgcc    3780
Ft_TypeB_LVS                 tatggtgtagcaggtgattttgatacagatggtcatgttgataacgttgcttgctttgcc    3780
Ft_TypeAI_SCHU_S4            tatggtgtagcaggtgattttgatacagatggtcgtgttgataacgttgcttgctttgcc    3778
Ft_TypeAI_MA00-2987          tatggtgtagcaggtgattttgatacagatggtcgtgttgataacgttgcttgctttgcc    3778
Ft_TypeAII_WY96-3418         tatggtgtagcaggtgattttgatacagatggtcatgttgataacgttgcttgctttgcc    3779
Ft_TypeAII_WY-00W4114        tatggtgtagcaggtgattttgatacagatggtcatgttgataacgttgcttgctttgcc    3779
                             ********************************** *************************

Ft_TypeB_FSC200              aataaaaatacgataattattcaaagttgttatgatgaaaatgatgagaactttgcacgc    3840
Ft_TypeB_LVS                 aataaaaatacgataattattcaaagttgttatgatgaaaatgatgagaactttgcacgc    3840
Ft_TypeAI_SCHU_S4            aataaaaatacgataattattcaaagttgttatgatgaaaatgatgagaactttgcacgc    3838
Ft_TypeAI_MA00-2987          aataaaaatacgataattattcaaagttgttatgatgaaaatgatgagaactttgcacgc    3838
Ft_TypeAII_WY96-3418         aataaaaatacgataattattcaaagttgttatgatgaaaatgatgagaactttgcacgc    3839
Ft_TypeAII_WY-00W4114        aataaaaatacgataattattcaaagttgttatgatgaaaatgatgagaactttgcacgc    3839
                             ************************************************************
```

```
Ft_TypeB_FSC200          catcaagcgaatatgacatatttagacaaatatgccagtgagtttaatatagtcgaaata    3900
Ft_TypeB_LVS             catcaagcgaatatgacatatttagacaaatatgccagtgagtttaatatagtcgaaata    3900
Ft_TypeAI_SCHU_S4        catcaagcgaatatgacatatttagacaaatatgccagtgagtttaatatagtcgaaata    3898
Ft_TypeAI_MA00-2987      catcaagcgaatatgacatatttagacaaatatgccagtgagtttaatatagtcgaaata    3898
Ft_TypeAII_WY96-3418     catcaagcgaatatgacatatttagacaaatatgccagtgagtttaatatagtcgaaata    3899
Ft_TypeAII_WY-00W4114    catcaagcgaatatgacatatttagacaaatatgccagtgagtttaatatagtcgaaata    3899
                         ************************************************************

Ft_TypeB_FSC200          cctcagcctcgagcacaatatttcgctggtgaacgattggctttatcttaccttaatttt    3960
Ft_TypeB_LVS             cctcagcctcgagcacaatatttcgctggtgaacgattggctttatcttaccttaatttt    3960
Ft_TypeAI_SCHU_S4        cctcagcctcgagcacaatatttcgctggtgaacgattggctttatcttaccttaatttt    3958
Ft_TypeAI_MA00-2987      cctcagcctcgagcacaatatttcgctggtgaacgattggctttatcttaccttaatttt    3958
Ft_TypeAII_WY96-3418     cctcagcctcgagcaaaatatttcgctggtgaacgattggctttatcttaccttaatttt    3959
Ft_TypeAII_WY-00W4114    cctcagcctcgagcaaaatatttcgctggtgaacgattggctttatcttaccttaatttt    3959
                         *************** ********************************************

Ft_TypeB_FSC200          tatattgtcaataacgctataattatgccagcatttggcgatccaaatgatgtcttatag    4020
Ft_TypeB_LVS             tatattgtcaataacgctataattatgccagcatttggcgatccaaatgatgtcttatag    4020
Ft_TypeAI_SCHU_S4        tatattgtcaataacgctataattatgccagcatttggcgatccaaatgatgtaat----    4014
Ft_TypeAI_MA00-2987      tatattgtcaataacgctataattatgccagcatttggcgatccaaatgatgtaat----    4014
Ft_TypeAII_WY96-3418     tatattgtcaataacgctataattatgccagcatttggcgatccaaatgatgtaat----    4015
Ft_TypeAII_WY-00W4114    tatattgtcaataacgctataattatgccagcatttggcgatccaaatgatgtaat----    4015
                         *****************************************************  *

Ft_TypeB_FSC200          ttaatccgagagtattttgttaaacacataagagatagcatcacaaaatttttcaaaact    4080
Ft_TypeB_LVS             ttaatccgagagtattttgttaaacacataagagatagcatcacaaaatttttcaaaact    4080
Ft_TypeAI_SCHU_S4        ------------------------------------------------------------    4014
Ft_TypeAI_MA00-2987      ------------------------------------------------------------    4014
Ft_TypeAII_WY96-3418     ------------------------------------------------------------    4015
Ft_TypeAII_WY-00W4114    ------------------------------------------------------------    4015

Ft_TypeB_FSC200          attattcacttttctaaatatttttttaaagttagcccaaaccttttcaataggatttaa    4140
Ft_TypeB_LVS             attattcacttttctaaatatttttttaaagttagcccaaaccttttcaataggatttaa    4140
Ft_TypeAI_SCHU_S4        ------------------------------------------------------------    4014
Ft_TypeAI_MA00-2987      ------------------------------------------------------------    4014
Ft_TypeAII_WY96-3418     ------------------------------------------------------------    4015
Ft_TypeAII_WY-00W4114    ------------------------------------------------------------    4015
```

```
Ft_TypeB_FSC200            atctggagagtacggaggtagatataatatttgtacatcaaatttattggctatttcaat    4200
Ft_TypeB_LVS               atctggagagtacggaggtagatataatatttgtacatcaaatttattggctatttcaat    4200
Ft_TypeAI_SCHU_S4          ------------------------------------------------------------    4014
Ft_TypeAI_MA00-2987        ------------------------------------------------------------    4014
Ft_TypeAII_WY96-3418       ------------------------------------------------------------    4015
Ft_TypeAII_WY-00W4114      ------------------------------------------------------------    4015

Ft_TypeB_FSC200            cagcttagaggatttatggaaactagcattatccattactatagtagttttaggttttaa    4260
Ft_TypeB_LVS               cagcttagaggatttatggaaactagcattatccattactatagtagttttaggttttaa    4260
Ft_TypeAI_SCHU_S4          ------------------------------------------------------------    4014
Ft_TypeAI_MA00-2987        ------------------------------------------------------------    4014
Ft_TypeAII_WY96-3418       ------------------------------------------------------------    4015
Ft_TypeAII_WY-00W4114      ------------------------------------------------------------    4015

Ft_TypeB_FSC200            tgatgggcataagtgttcctcaaaccattgattaaaaatttcagtattggtatatccgct    4320
Ft_TypeB_LVS               tgatgggcataagtgttcctcaaaccattgattaaaaatttcagtattggtatatccgct    4320
Ft_TypeAI_SCHU_S4          ------------------------------------------------------------    4014
Ft_TypeAI_MA00-2987        ------------------------------------------------------------    4014
Ft_TypeAII_WY96-3418       ------------------------------------------------------------    4015
Ft_TypeAII_WY-00W4114      ------------------------------------------------------------    4015

Ft_TypeB_FSC200            gtactctaatggagctataatctttttatctgcataattatatccagcaacaatacttct    4380
Ft_TypeB_LVS               gtactctaatggagctataatctttttatctgcataattatatccagcaacaatacttct    4380
Ft_TypeAI_SCHU_S4          ------------------------------------------------------------    4014
Ft_TypeAI_MA00-2987        ------------------------------------------------------------    4014
Ft_TypeAII_WY96-3418       ------------------------------------------------------------    4015
Ft_TypeAII_WY-00W4114      ------------------------------------------------------------    4015

Ft_TypeB_FSC200            tctttgtgtttgatatgctaaaacctcgccataactaggctcaccaattagtgaccatcc    4440
Ft_TypeB_LVS               tctttgtgtttgatatgctaaaacctcgccataactaggctcaccaattagtgaccatcc    4440
Ft_TypeAI_SCHU_S4          ------------------------------------------------------------    4014
Ft_TypeAI_MA00-2987        ------------------------------------------------------------    4014
Ft_TypeAII_WY96-3418       ------------------------------------------------------------    4015
Ft_TypeAII_WY-00W4114      ------------------------------------------------------------    4015
```

```
Ft_TypeB_FSC200            tcttaggatagaaagcttattgtcacaccacatctcatctatataaaataacaagttttg    4500
Ft_TypeB_LVS               tcttaggatagaaagcttattgtcacaccacatctcatctatataaaataacaagttttg    4500
Ft_TypeAI_SCHU_S4          ------------------------------------------------------------    4014
Ft_TypeAI_MA00-2987        ------------------------------------------------------------    4014
Ft_TypeAII_WY96-3418       ------------------------------------------------------------    4015
Ft_TypeAII_WY-00W4114      ------------------------------------------------------------    4015

Ft_TypeB_FSC200            agctatttcttttagtttttctatatactccaacctttcatgttcttttctttgcttata    4560
Ft_TypeB_LVS               agctatttcttttagtttttctatatactccaacctttcatgttcttttctttgcttata    4560
Ft_TypeAI_SCHU_S4          ------------------------------------------------------------    4014
Ft_TypeAI_MA00-2987        ------------------------------------------------------------    4014
Ft_TypeAII_WY96-3418       ------------------------------------------------------------    4015
Ft_TypeAII_WY-00W4114      ------------------------------------------------------------    4015

Ft_TypeB_FSC200            ttttggagtctttttttaaaactaaaaccaagtatattaagacaatcataaaatgtactt    4620
Ft_TypeB_LVS               ttttggagtctttttttaaaactaaaaccaagtatattaagacaatcataaaatgtactt    4620
Ft_TypeAI_SCHU_S4          ------------------------------------------------------------    4014
Ft_TypeAI_MA00-2987        ------------------------------------------------------------    4014
Ft_TypeAII_WY96-3418       ------------------------------------------------------------    4015
Ft_TypeAII_WY-00W4114      ------------------------------------------------------------    4015

Ft_TypeB_FSC200            cttggaatatcaggggctaatgcttcttttatatctaatgtacttgcatctggatgatct    4680
Ft_TypeB_LVS               cttggaatatcaggggctaatgcttcttttatatctaatgtacttgcatctggatgatct    4680
Ft_TypeAI_SCHU_S4          ------------------------------------------------------------    4014
Ft_TypeAI_MA00-2987        ------------------------------------------------------------    4014
Ft_TypeAII_WY96-3418       ------------------------------------------------------------    4015
Ft_TypeAII_WY-00W4114      ------------------------------------------------------------    4015

Ft_TypeB_FSC200            atcaaatactgttcaatcaatgttttatcggtaaagctagcgactctgccacaaccaact    4740
Ft_TypeB_LVS               atcaaatactgttcaatcaatgttttatcggtaaagctagcgactctgccacaaccaact    4740
Ft_TypeAI_SCHU_S4          ------------------------------------------------------------    4014
Ft_TypeAI_MA00-2987        ------------------------------------------------------------    4014
Ft_TypeAII_WY96-3418       ------------------------------------------------------------    4015
Ft_TypeAII_WY-00W4114      ------------------------------------------------------------    4015
```

```
Ft_TypeB_FSC200             ccttgccttgaactataatctccggttcttttataaagctctatccatgaaacaactgta     4800
Ft_TypeB_LVS                ccttgccttgaactataatctccggttcttttataaagctctatccatgaaacaactgta     4800
Ft_TypeAI_SCHU_S4           ------------------------------------------------------------     4014
Ft_TypeAI_MA00-2987         ------------------------------------------------------------     4014
Ft_TypeAII_WY96-3418        ------------------------------------------------------------     4015
Ft_TypeAII_WY-00W4114       ------------------------------------------------------------     4015

Ft_TypeB_FSC200             cgcttatctatgttaaaaaacttactcagctcgaactccgtcataccttcttcatattta     4860
Ft_TypeB_LVS                cgcttatctatgttaaaaaacttactcagctcgaactccgtcataccttcttcatattta     4860
Ft_TypeAI_SCHU_S4           ------------------------------------------------------------     4014
Ft_TypeAI_MA00-2987         ------------------------------------------------------------     4014
Ft_TypeAII_WY96-3418        ------------------------------------------------------------     4015
Ft_TypeAII_WY-00W4114       ------------------------------------------------------------     4015

Ft_TypeB_FSC200             ttaattacgatgtctctaaaatcttggctatatgatggcatttttattagacattataac     4920
Ft_TypeB_LVS                ttaattacgatgtctctaaaatcttggctatatgatggcatttttattagacattataac     4920
Ft_TypeAI_SCHU_S4           ------------------------------------------------------------     4014
Ft_TypeAI_MA00-2987         ------------------------------------------------------------     4014
Ft_TypeAII_WY96-3418        ------------------------------------------------------------     4015
Ft_TypeAII_WY-00W4114       ------------------------------------------------------------     4015

Ft_TypeB_FSC200             atttctacaaatatctttttctacaaatatctttcggattaactatagcttttgaaattc     4980
Ft_TypeB_LVS                atttctacaaatatctttttctacaaatatctttcggattaactatagcttttgaaattc     4980
Ft_TypeAI_SCHU_S4           ----------------------------------------------agcttttgaaattc     4028
Ft_TypeAI_MA00-2987         ----------------------------------------------agcttttgaaattc     4028
Ft_TypeAII_WY96-3418        ----------------------------------------------agcttttgaaattc     4029
Ft_TypeAII_WY-00W4114       ----------------------------------------------agcttttgaaattc     4029
                                                                          **************

Ft_TypeB_FSC200             tacagaaatgttttaaggatcgtagtattgaacagcttaatattattgaccttgtaattg     5040
Ft_TypeB_LVS                tacagaaatgttttaaggatcgtagtattgaacagcttaatattattgaccttgtaattg     5040
Ft_TypeAI_SCHU_S4           tacagaaatgttttaaggatcgtagtattgaacagcttaatattattgaccttgtaattg     4088
Ft_TypeAI_MA00-2987         tacagaaatgttttaaggatcgtagtattgaacagcttaatattattgaccttgtaattg     4088
Ft_TypeAII_WY96-3418        tacagaaatgttttaaggatcgtagtattgaacagcttaatattattgaccttgtaattg     4089
Ft_TypeAII_WY-00W4114       tacagaaatgttttaaggatcgtagtattgaacagcttaatattattgaccttgtaattg     4089
                            ************************************************************
```

```
Ft_TypeB_FSC200              gtggaggaggaattcactgtattactatgcaacaaccggctataaaggagatttgatatg   5100
Ft_TypeB_LVS                 gtggaggaggaattcactgtattactatgcaacaaccggctataaaggagatttgatatg   5100
Ft_TypeAI_SCHU_S4            gtggaggaggaattcactgtattactatgcaacaaccggctataaaggagatttgatatg   4148
Ft_TypeAI_MA00-2987          gtggaggaggaattcactgtattactatgcaacaaccggctataaaggagatttgatatg   4148
Ft_TypeAII_WY96-3418         gtggaggaggaattcactgtattactatgcaacaaccggctataaaggagatttgatatg   4149
Ft_TypeAII_WY-00W4114        gtggaggaggaattcactgtattactatgcaacaaccggctataaaggagatttgatatg   4149
                             ************************************************************

Ft_TypeB_FSC200              gcgaatataaaagttgcagttgtccaattatcttttaatgataatgaagctgaaaattta   5160
Ft_TypeB_LVS                 gcgaatataaaagttgcagttgtccaattatcttttaatgataatgaagctgaaaattta   5160
Ft_TypeAI_SCHU_S4            gcgaatataaaagttgcagttgtccaattatcttttaatgataatgaagctgaaaattta   4208
Ft_TypeAI_MA00-2987          gcgaatataaaagttgcagttgtccaattatcttttaatgataatgaagctgaaaattta   4208
Ft_TypeAII_WY96-3418         gcgaatataaaagttgcagttgtccaattatcttttaatgataatgaagctgaaaattta   4209
Ft_TypeAII_WY-00W4114        gcgaatataaaagttgcagttgtccaattatcttttaatgataatgaagctgaaaattta   4209
                             ************************************************************

Ft_TypeB_FSC200              gcaaaactggagagtaaaattattcaagcagctaaaaatggtgcaaaaataattcttacc   5220
Ft_TypeB_LVS                 gcaaaactggagagtaaaattattcaagcagctaaaaatggtgcaaaaataattcttacc   5220
Ft_TypeAI_SCHU_S4            gcaaaactggagagtaaaattattcaagcagctaaaaatggtgcaaaaataattcttact   4268
Ft_TypeAI_MA00-2987          gcaaaactggagagtaaaattattcaagcagctaaaaatggtgcaaaaataattcttact   4268
Ft_TypeAII_WY96-3418         gcaaaactggagagtaaaattattcaagcagctaaaaatggtgcaaaaataattcttact   4269
Ft_TypeAII_WY-00W4114        gcaaaactggagagtaaaattattcaagcagctaaaaatggtgcaaaaataattcttact   4269
                             ***********************************************************

Ft_TypeB_FSC200              ccagagttaccaagttatctatatttttgcaaaaaataaaattctaaatattttgattta   5280
Ft_TypeB_LVS                 ccagagttaccaagttatctatatttttgcaaaaaataaaattctaaatattttgattta   5280
Ft_TypeAI_SCHU_S4            ccagagttaccaagttatctatatttttgcaaaaaacaaaattctaaatattttgattta   4328
Ft_TypeAI_MA00-2987          ccagagttaccaagttatctatatttttgcaaaaaacaaaattctaaatattttgattta   4328
Ft_TypeAII_WY96-3418         ccagagttaccaagttatctatatttttgcaaaaaacaaaattctaaatattttgattta   4329
Ft_TypeAII_WY-00W4114        ccagagttaccaagttatctatatttttgcaaaaaacaaaattctaaatattttgattta   4329
                             ************************************ ***********************

Ft_TypeB_FSC200              gctaaaaccattgatgaatcaccaatagtaaaattatataaactcttagcacataaatat   5340
Ft_TypeB_LVS                 gctaaaaccattgatgaatcaccaatagtaaaattatataaactcttagcacataaatat   5340
Ft_TypeAI_SCHU_S4            gctaaaaccattgatgaatcaccaatagtaaaattatataaactcttagcacataaatat   4388
Ft_TypeAI_MA00-2987          gctaaaaccattgatgaatcaccaatagtaaaattatataaactcttagcacataaatat   4388
Ft_TypeAII_WY96-3418         gctaaaaccattgatgaatcaccaatagtaaaattatataaactcttagcacataaatat   4389
Ft_TypeAII_WY-00W4114        gctaaaaccattgatgaatcaccaatagtaaaattatataaactcttagcacataaatat   4389
                             ************************************************************
```

```
Ft_TypeB_FSC200            aatattgttttgcctgctagtttttttgagagagatggaaatgcttgttataactcgata   5400
Ft_TypeB_LVS               aatattgttttgcctgctagtttttttgagagagatggaaatgcttgttataactcgata   5400
Ft_TypeAI_SCHU_S4          aatattgttttgcctgctagtttttttgagagagatggaaatgcttgttataactcgata   4448
Ft_TypeAI_MA00-2987        aatattgttttgcctgctagtttttttgagagagatggaaatgcttgttataactcgata   4448
Ft_TypeAII_WY96-3418       aatattgttttgcctgctagtttttttgagagagatggaaatgcttgttataactcgata   4449
Ft_TypeAII_WY-00W4114      aatattgttttgcctgctagtttttttgagagagatggaaatgcttgttataactcgata   4449
                           ************************************************************

Ft_TypeB_FSC200            gcaatgattgatgcggatggttcgataatgggtatatatcgtaaagcccatattccagac   5460
Ft_TypeB_LVS               gcaatgattgatgcggatggttcgataatgggtatatatcgtaaagcccatattccagac   5460
Ft_TypeAI_SCHU_S4          gcaatgattgatgcggatggttcgataatgggtgtatatcgtaaagcccatattccagac   4508
Ft_TypeAI_MA00-2987        gcaatgattgatgcggatggttcgataatgggtgtatatcgtaaagcccatattccagac   4508
Ft_TypeAII_WY96-3418       gcaatgattgatgcggatggttcgataatgggtgtatatcgtaaagcccatattccagac   4509
Ft_TypeAII_WY-00W4114      gcaatgattgatgcggatggttcgataatgggtgtatatcgtaaagcccatattccagac   4509
                           ******************************** ***************************

Ft_TypeB_FSC200            ggtattggttaccaagagaaatattatttctcacctggaagtgttggttttaaggtttag   5520
Ft_TypeB_LVS               ggtattggttaccaagagaaatattatttctcacctggaagtgttggttttaaggtttag   5520
Ft_TypeAI_SCHU_S4          ggtattggttaccaagagaaatattatttctcacctggaagtgctggttttaaggtttgg   4568
Ft_TypeAI_MA00-2987        ggtattggttaccaagagaaatattatttctcacctggaagtgctggttttaaggtttgg   4568
Ft_TypeAII_WY96-3418       ggtattggttaccaagagaaatattatttctcacctggaagtgctggttttaaggtttgg   4569
Ft_TypeAII_WY-00W4114      ggtattggttaccaagagaaatattatttctcacctggaagtgctggttttaaggtttgg   4569
                           ******************************************* ************** *

Ft_TypeB_FSC200            gatactaaatatgctaaagttggagttggtatttgctgggatcaatggtttccagaagct   5580
Ft_TypeB_LVS               gatactaaatatgctaaagttggagttggtatttgctgggatcaatggtttccagaagct   5580
Ft_TypeAI_SCHU_S4          gatactaaatatgctaaagttggagttggtatttgctgggatcaatggtttccagaagct   4628
Ft_TypeAI_MA00-2987        gatactaaatatgctaaagttggagttggtatttgctgggatcaatggtttccagaagct   4628
Ft_TypeAII_WY96-3418       gatactaaatatgctaaagttggagttggtatttgctgggatcaatggtttccagaagct   4629
Ft_TypeAII_WY-00W4114      gatactaaatatgctaaagttggagttggtatttgctgggatcaatggtttccagaagct   4629
                           ************************************************************

Ft_TypeB_FSC200            gctagagtaatggctttaaaaggtgctgaaattttattatatccaacagcaatagggagc   5640
Ft_TypeB_LVS               gctagagtaatggctttaaaaggtgctgaaattttattatatccaacagcaatagggagc   5640
Ft_TypeAI_SCHU_S4          gctagagtaatggctttaaaaggtgctgaaattttattatatccaacagcaatagggagc   4688
Ft_TypeAI_MA00-2987        gctagagtaatggctttaaaaggtgctgaaattttattatatccaacagcaatagggagc   4688
Ft_TypeAII_WY96-3418       gctagagtaatggctttaaaaggtgctgaaattttattatatccaacagcaataggaagc   4689
Ft_TypeAII_WY-00W4114      gctagagtaatggctttaaaaggtgctgaaattttattatatccaacagcaatagggagc   4689
                           ******************************************************** ***
```

```
Ft_TypeB_FSC200              gaactacacttaccagattacgattcaaaagatcattggcaaagagtgatgcaagggcat    5700
Ft_TypeB_LVS                 gaactacacttaccagattacgattcaaaagatcattggcaaagagtgatgcaagggcat    5700
Ft_TypeAI_SCHU_S4            gaaccacacttaccagattacgattcaaaagatcattggcaaagagtgatgcaagggcat    4748
Ft_TypeAI_MA00-2987          gaaccacacttaccagattacgattcaaaagatcattggcaaagagtgatgcaagggcat    4748
Ft_TypeAII_WY96-3418         gaaccacacttaccagattacgattcaaaagatcattggcaaagagtgatgcaagggcat    4749
Ft_TypeAII_WY-00W4114        gaaccacacttaccagattacgattcaaaagatcattggcaaagagtgatgcaagggcat    4749
                             **** *******************************************************

Ft_TypeB_FSC200              gctgcggtaaatatgttgcccgtattagcatcaaatagatatgcaactgaggcaaatgat    5760
Ft_TypeB_LVS                 gctgcggtaaatatgttgcccgtattagcatcaaatagatatgcaactgaggcaaatgat    5760
Ft_TypeAI_SCHU_S4            gctgcggcaaatatgttgcccgtattagcatcaaatagatatgcaactgaggcaaatgat    4808
Ft_TypeAI_MA00-2987          gctgcggcaaatatgttgcccgtattagcatcaaatagatatgcaactgaggcaaatgat    4808
Ft_TypeAII_WY96-3418         gctgcggcaaatatgttgcccgtattagcatcaaatagatatgcaactgaggcaaatgat    4809
Ft_TypeAII_WY-00W4114        gctgcggcaaatatgttgcccgtattagcatcaaatagatatgcaactgaggcaaatgat    4809
                             ******* ****************************************************

Ft_TypeB_FSC200              aatatcacagcaacttattatggcagctcattcataactgatcatactggtgataaaatt    5820
Ft_TypeB_LVS                 aatatcacagcaacttattatggcagctcattcataactgatcatactggtgataaaatt    5820
Ft_TypeAI_SCHU_S4            gatatcacagcaacttattatggcagctcattcataactgatcatactggtgataaaatt    4868
Ft_TypeAI_MA00-2987          gatatcacagcaacttattatggcagctcattcataactgatcatactggtgataaaatt    4868
Ft_TypeAII_WY96-3418         gatatcacagcaacttattatggcagctcattcataactgatcatactggtgataaaatt    4869
Ft_TypeAII_WY-00W4114        gatatcacagcaacttattatggcagctcattcataactgatcatactggtgataaaatt    4869
                              ***********************************************************

Ft_TypeB_FSC200              gctgaggctgacagaagcgatgacgatatactttatgcaacatttgattttgctgaacta    5880
Ft_TypeB_LVS                 gctgaggctgacagaagcgatgacgatatactttatgcaacatttgattttgctgaacta    5880
Ft_TypeAI_SCHU_S4            gctgaggctgacagaagcggtgacgatatactttatgcaacatttgattttgctgaacta    4928
Ft_TypeAI_MA00-2987          gctgaggctgacagaagcggtgacgatatactttatgcaacatttgattttgctgaacta    4928
Ft_TypeAII_WY96-3418         gctgaggctgacagaagcggtgacgatatactttatgcaacatttgattttgctgaacta    4929
Ft_TypeAII_WY-00W4114        gctgaggctgacagaagcggtgacgatatactttatgcaacatttgattttgctgaacta    4929
                             ******************* ****************************************

Ft_TypeB_FSC200              cagcagcaaaggttttattggggattatttagggatcgtcgccctgagctttatgatgaa    5940
Ft_TypeB_LVS                 cagcagcaaaggttttattggggattatttagggatcgtcgccctgagctttatgatgaa    5940
Ft_TypeAI_SCHU_S4            cagcagcaaaggttttattggggattatttagggatcgtcgccctgagctttatgatgaa    4988
Ft_TypeAI_MA00-2987          cagcagcaaaggttttattggggattatttagggatcgtcgccctgagctttatgatgaa    4988
Ft_TypeAII_WY96-3418         cagcagcaaaggttttattggggattatttagggatcgtcgccctgagctttatgatgaa    4989
Ft_TypeAII_WY-00W4114        cagcagcaaaggttttattggggattatttagggatcgtcgccctgagctttatgatgaa    4989
                             ************************************************************
```

```
Ft_TypeB_FSC200              attgttagaaagtattaaattgataaaaatgtttttagtttagagttctaacctgcataa    6000
Ft_TypeB_LVS                 attgttagaaagtattaaattgataaaaatgtttttagtttagagttctaacctgcataa    6000
Ft_TypeAI_SCHU_S4            attgttagaaagtattaaattgataaaaatgtttttagtttagagctctaacctgcataa    5048
Ft_TypeAI_MA00-2987          attgttagaaagtattaaattgataaaaatgtttttagtttagagctctaacctgcataa    5048
Ft_TypeAII_WY96-3418         attgttagaaagtattaaattgataaaaatgtttttagtttagagttctaacctgcataa    5049
Ft_TypeAII_WY-00W4114        attgttagaaagtattaaattgataaaaatgtttttagtttagagttctaacctgcataa    5049
                             ********************************************* **************
```

SUPPLEMENTARY MATERIAL

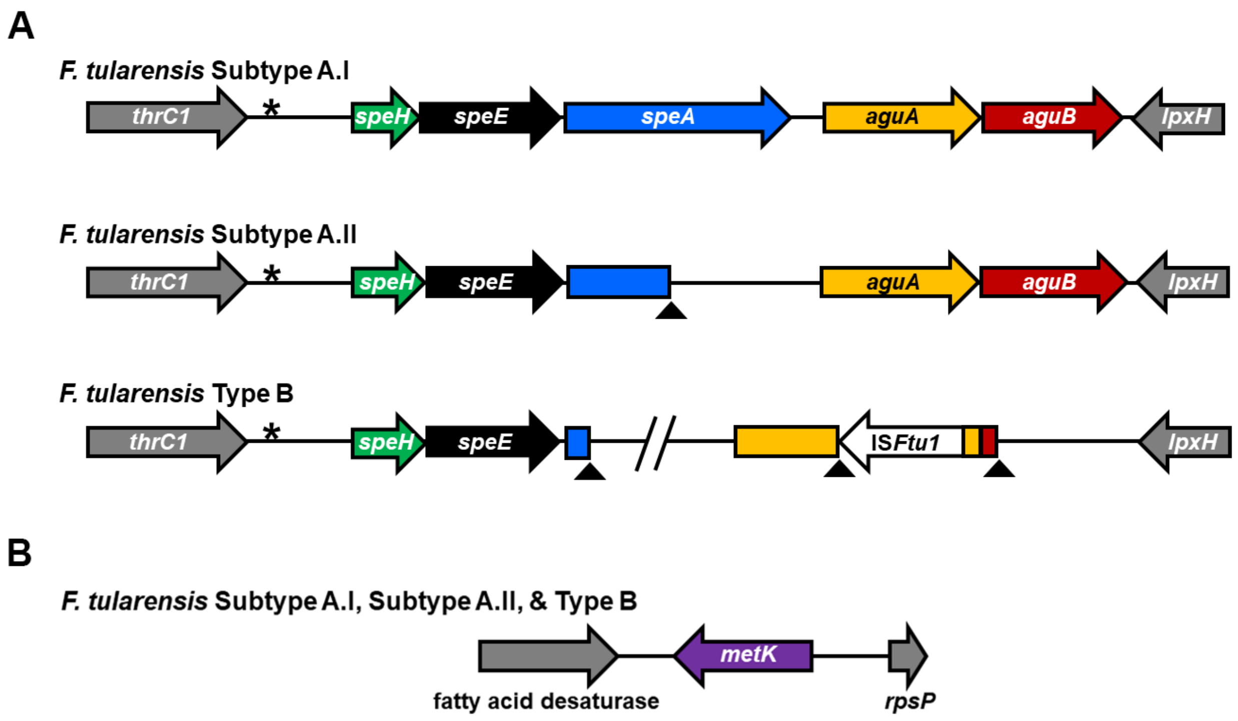

Figure S2. Comparison of the genes 5' and 3' to the *speHEA* operon, *aguAB* operon, and *metK* within the genomes of the *F. tularensis* subtype A.I, subtype A.II, and type B clades. **(A)** The threonine synthase (*thrC1*) gene was adjacent and 5' to *speH* while UDP-2,3-diacylglucosamine hydrolase (*lpxH*) gene was adjacent and 3' to *aguB*. The remnant of a beta-galactosidase gene encoding only the first 76 amino acids of the 656-residue full-length enzyme is denoted with an asterisk. **(B)** Fatty acid desaturase gene was located 5' to *metK* and the 30S ribosomal protein S16 (*rpsP*) gene was positioned 3' to *metK*. In panel A, arrowheads denote a premature stop codon that results in a truncated gene.

SUPPLEMENTARY MATERIAL

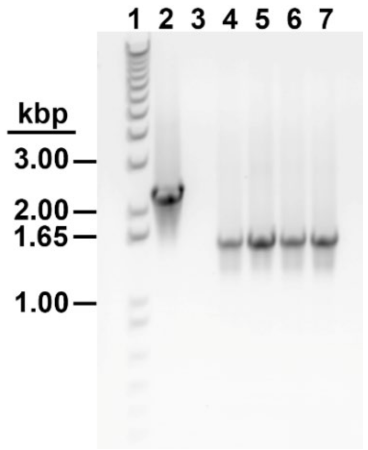

Figure S3. PCR amplification of *speHEA* chromosomal locus in *F. tularensis* wild-type LVS and the isogenic Δ*speE* mutants. Shown are *F. tularensis* wild-type LVS (lane 2) and the LVS Δ*speE* mutant used for this study (lane 4). Three additional LVS Δ*speE* mutants (lanes 5, 6, and 7), the no template control (lane 3), and a DNA marker (lane 1) are also shown.

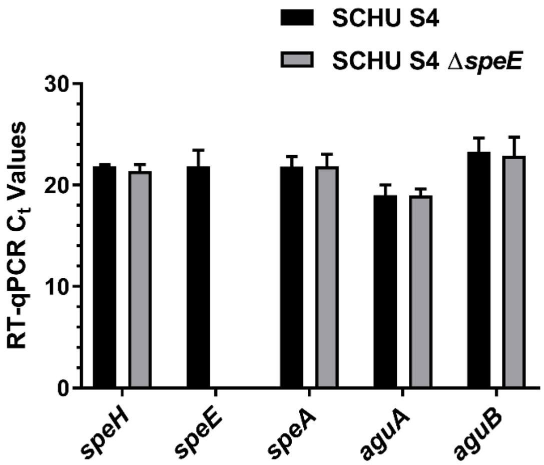

Figure S4. Reverse transcription quantitative real-time PCR (RT-qPCR) of *speHEA* and *aguAB* in *F. tularensis* wild-type SCHU S4 and the isogenic Δ*speE* mutant. *F. tularensis* strains were cultured in brain heart infusion broth (BHI) to mid-exponential growth phase prior to RNA isolation, DNase treatment, and cDNA synthesis for RT-qPCR. Crossing threshold (C_t) values are shown and were normalized to *lpnA* mRNA. Mean with ± SEM is shown for triplicate RT-qPCR samples in three independent experiments. Data were analyzed using the paired nonparametric t-test, and the C_t values obtained for these transcribed genes were not significant ($P > 0.05$) between the wildtype and mutant.
